# KDM5B recruits FOXG1 to suppress the IFN response, leading to malignant progression and immune evasion in cervical cancer

**DOI:** 10.3389/fmed.2026.1749536

**Published:** 2026-04-01

**Authors:** Guangping He, Jiali Liang, Ziyang Ding, Xinyu Qu, Junjun Qiu

**Affiliations:** 1 Obstetrics and Gynecology Hospital of Fudan University, Shanghai, China; 2 Shanghai Key Laboratory of Female Reproductive Endocrine Related Diseases, Shanghai, China; 3 Shanghai Key Laboratory of Reproduction and Development, Shanghai, China

**Keywords:** cervical cancer, DNA damage, FOXG1, IFN, immune escape, KDM5B

## Abstract

**Introduction:**

Cervical cancer carcinogenesis often exhibits significant heterogeneity, and the molecular mechanisms underlying epithelial cell transformation are not fully understood.

**Methods:**

Single-cell RNA sequencing was employed to compare the immune microenvironment of normal cervix and cervical cancer. Immunohistochemistry was applied to measure the expression of KDM5B in patient samples. qPCR and western blot were performed to detect the KDM5B expression and activation of IFN pathway in cell line. Co-immunoprecipitation (COIP), immunofluorescence (IF) and surface plasmon resonance (SPR) are employed to detect the binding between KDM5B and FOXG1. Flow cytometry was conducted to evaluate the immune infiltration of mice tumor.

**Results:**

This study reveals that malignant progression in cervical cancer is characterized by substantial DNA damage and marked upregulation of the histone demethylase KDM5B. We also identified a novel regulatory mechanism by which KDM5B recruits the transcriptional repressor FOXG1 to suppress the interferon signaling pathway, thereby promoting malignant behaviors and immune escape in cervical cancer. Targeting KDM5B could effectively enhance anti-tumor immunity.

**Discussion:**

These findings provide crucial insights into the biological role of KDM5B in cervical cancer and highlight its potential as a promising therapeutic target for immunotherapy.

## Introduction

Cervical cancer remains the fourth most common cancer among women worldwide, with approximately 660,000 new cases reported in 2022, imposing a substantial burden on global public health (1). Although treatment strategies, including surgery, radiotherapy, chemotherapy, and immunotherapy, have continued to advance, a significant number of patients still experience drug resistance, disease recurrence, or metastasis, resulting in poor prognosis (2). The emergence of precision medicine and rapid developments in technologies such as single-cell sequencing have progressively enhanced our understanding of the complexity of cervical cancer (3). Nevertheless, the core biological processes of cervical carcinogenesis, specifically the malignant transformation of epithelial cells, remain incompletely elucidated.

DNA damage and repair represent one of the most critical processes in the malignant transformation of epithelial cells (4). DNA damage often leads to genomic instability (5). When such damage persists and DNA repair mechanisms become compromised, gene mutations accumulate progressively (6). This process accelerates the activation of oncogenes and the inactivation of tumor suppressor genes, enabling cells to acquire uncontrolled proliferative capacity, evade apoptosis, and enhance invasiveness (7). However, in the context of cervical carcinogenesis, the specific role of DNA damage remains unclear, underscoring the need to further explore the underlying mechanisms of cervical cancer development.

KDM5B is a classical histone-modifying enzyme and a member of the JMJD family (8). It is frequently upregulated in multiple types of tumors (9). By catalyzing the demethylation of H3K4me2/3, KDM5B represses the expression of various tumor suppressor genes and promotes tumor progression by regulating key biological processes such as the cell cycle and apoptosis (10, 11). Moreover, KDM5B has been implicated in tumor immune evasion, making it a potential target for cancer immunotherapy (12). However, its role in cervical cancer remains unclear, and whether it regulates gene expression through other mechanisms remains to be determined, underscoring the importance of further investigating the crucial functions of this molecule in cervical cancer cells.

In this study, we observed that cervical cancer cells undergo substantial DNA damage during malignant transformation, accompanied by a marked upregulation of KDM5B. By investigating the specific mechanism through which KDM5B influences cervical cancer progression and immune evasion, we found that KDM5B suppresses IFN signaling by recruiting FOXG1. Notably, targeting KDM5B can inhibit tumor growth, activate antitumor responses, and enhance the efficacy of anti-PD1 therapy. Our findings elucidate the biological alterations during cervical carcinogenesis and demonstrate the regulatory role of KDM5B in cervical cancer, highlighting it as a potential therapeutic target.

## Materials and methods

### Cell lines and tumor injections

All cell lines were mycoplasma-free and cultured at 37 °C with 5% CO₂. TC1 cells were maintained in RPMI-1640 (Gibco), while SiHa cells were grown in DMEM (Gibco), both supplemented with 10% FBS (Gibco). For tumor induction, TC1 cells were washed twice with 1 × PBS, resuspended in PBS (Servicebio), and subcutaneously injected into mice (3 × 10^5^ cells per mouse).

### *In vivo* experiments

Female C57BL/6 mice (6–8 weeks old) were obtained from SPF Biotech and maintained under specific pathogen-free (SPF) conditions throughout the study period. Tumor growth was monitored by a caliper. When TC1 tumor volumes reached approximately 100 mm^3^, mice were randomized into different treatment groups (*n =* 5 per group). KDM5B inhibitor (MCE) was used at 30 mg/kg administered daily via intraperitoneal injection. Anti-PD1 antibody (Bioxcell) was administered intraperitoneally to each animal at a dose of 200 μg once every 3 days.

Tumor volumes were calculated as 0.5 × length × width^2^. Animals were continuously monitored, and mice were euthanized via CO₂ asphyxiation when any of the following endpoints were met: study termination, tumor burden ≥2,000 mm^3^, ulceration, >20% body weight loss, or moribund appearance. CO₂ was delivered at a rate of 10–30% of the chamber volume per minute. After loss of consciousness was confirmed, the flow rate was increased, ensuring that the pressure did not exceed 0.5 kPa.

### Re-analysis of the dataset from the public database

Patient characteristics and scRNA-seq of cervical cancer were downloaded from the Gene Expression Omnibus (GEO, accession GSE120575). Data processing, including quality control, normalization, and scaling, was performed using Seurat. Cluster identity was annotated based on the expression of canonical marker genes. Subsequently, differentially expressed genes (DEGs) between cell populations were identified using the Find All Markers function. Gene Ontology (GO) enrichment analyses were then performed on the DEGs to elucidate potential biological functions and signaling pathways associated with each cell population. Pseudotime trajectory analysis was performed using Monocle2 to reconstruct developmental dynamics and transitional relationships among epithelial cells and their gene expressions.

### Immunohistochemistry

Tumor specimens were fixed in 4% paraformaldehyde, processed into paraffin blocks, and sectioned. After deparaffinization and heat-induced antigen retrieval in sodium citrate buffer, endogenous peroxidases were quenched with hydrogen peroxide. Sections were blocked with bovine serum albumin (BSA) (Servicebio) and then incubated overnight at 4 °C with a primary antibody against KDM5B. Following washes, binding was detected using an HRP-conjugated secondary antibody with 3,3′-diaminobenzidine (DAB) (Servicebio) substrate, and the sections were mounted for microscopic evaluation.

### Immunofluorescence

Following PBS washes, cells were fixed with 4% formaldehyde, then permeabilized and blocked with 3% BSA. Subsequently, cells were incubated with primary antibodies (anti-KDM5B) (Proteintech) overnight at 4 °C, followed by fluorophore-conjugated secondary antibodies. After thorough washes, cells were incubated with the anti-FOXG1 antibody (Proteintech) labeled with red fluorescence overnight at 4 °C. The nuclei were then counterstained with DAPI (Servicebio), and the slides were mounted for fluorescence microscopy.

### qPCR

Cells were cultured to 70–80% confluence in 24-well plates for total RNA extraction using a commercial kit (EZB). RNA quality was verified using a NanoDrop spectrophotometer. Subsequently, 1 μg of RNA was reverse-transcribed into cDNA. Quantitative PCR was performed in triplicate using SYBR Green Master Mix (EZB) on an Applied Biosystems instrument, with GAPDH and *β*-actin as endogenous controls. Gene expression levels were quantified via the 2^(−ΔΔCt) method.

### Western blot

Cells were lysed in an ice-cold radio-immunoprecipitation assay (RIPA) buffer (Epizyme) containing protease and phosphatase inhibitors. After centrifugation, protein concentration was determined using a bicinchoninic acid (BCA) assay (Epizyme). Equal amounts of protein were separated by sodium dodecyl sulfate polyacrylamide gel electrophoresis (SDS-PAGE), transferred to polyvinylidene fluoride (PVDF) membranes (Merck), and blocked with 5% BSA. The membranes were sequentially incubated with primary antibodies overnight at 4 °C, followed by horseradish peroxidase (HRP)-conjugated secondary antibodies at room temperature. Protein bands were visualized using an enhanced chemiluminescence substrate.

### Co-immunoprecipitation

Cells were then lysed, and the clarified lysates were subjected to immunoprecipitation overnight at 4 °C using either an anti-KDM5B antibody or control IgG (Proteintech). Immune complexes were captured with Protein A/G magnetic beads (MCE), stringently washed, and prepared for Western blot analysis.

### Surface plasmon resonance (SPR)

SPR binding kinetics were analyzed on a BIAcore instrument at 25 °C. Recombinant KDM5B protein was immobilized on a CM5 chip (~11,000–16,000 RU). Analytes (FOXG1 protein) were injected at 30 μL/min with association and dissociation times of 90–120 s and 90–180 s, respectively. The surface was regenerated with 10 mM glycine–HCl (pH 2.0). Sensorgram data were processed using BIAcore Evaluation Software and fitted to a 1:1 Langmuir binding model to determine association (Ka) and dissociation (Kd) rate constants, from which equilibrium dissociation constants (KD) were derived as KD = Kd/Ka.

### Flow cytometry

Cells were stained with a fixable viability dye and Fc receptor block (BioLegend), followed by surface antigen labeling with fluorophore-conjugated antibodies. For intracellular targets, cells were treated with brefeldin A prior to fixation and permeabilization using a specialized buffer set (BioLegend). After intracellular staining, samples were acquired on an LSR Fortessa or CytoFlex flow cytometer. Compensation beads (BioLegend) and FMO controls were included to ensure accurate panel setup and gating. For CD8^+^ T cell analysis, cells were pre-gated on live, singlets, CD45^+^, CD3^+^, and CD8^+^ markers. For CD4^+^ T cell analysis, cells were pre-gated on live, singlets, CD45^+^, CD3^+^, and CD4^+^ markers.

### CCK-8 viability assay

The cells were seeded in 96-well plates (2,000 cells per well). At designated intervals, CCK-8 reagent (MCE) was added, and absorbance at 450 nm was measured using a microplate reader.

### Transwell experiment

SiHa cells were resuspended and seeded into the upper Transwell chamber at 5 × 10^5^ cells/mL in 100 μL of serum-free medium. The lower chamber was filled with 700 μL of complete medium containing 20% serum as a chemoattractant. After 48 h of incubation, the cells that had migrated were fixed, stained, and imaged under a microscope (10 × magnification). Quantification was performed using ImageJ software.

### Wound healing assay

In 6-well plates, cells were seeded at a density of 5 × 10^5^ cells per well and cultured until they reached confluence. A uniform scratch was created in each well using a 250-μL pipette tip. Subsequently, the culture medium was removed, and the cells were gently washed with 500 μL of PBS. Serum-free DMEM medium was then added to each well. Immediately after medium replacement, images of the scratch wounds were captured at 0 h and 48 h using an inverted microscope.

### Cell apoptosis detection

Cells were harvested by trypsinization, resuspended, and stained with 5 μL of Annexin V in the dark for 15 min at room temperature. Subsequently, 10 μL of propidium iodide (PI, 20 μg/mL) and 400 μL of PBS were added. Cell apoptosis was then immediately analyzed by flow cytometry.

## Results

### Single-cell sequencing reveals DNA damage and remodeling of epithelial cells in cervical cancer

**Figure 1:**
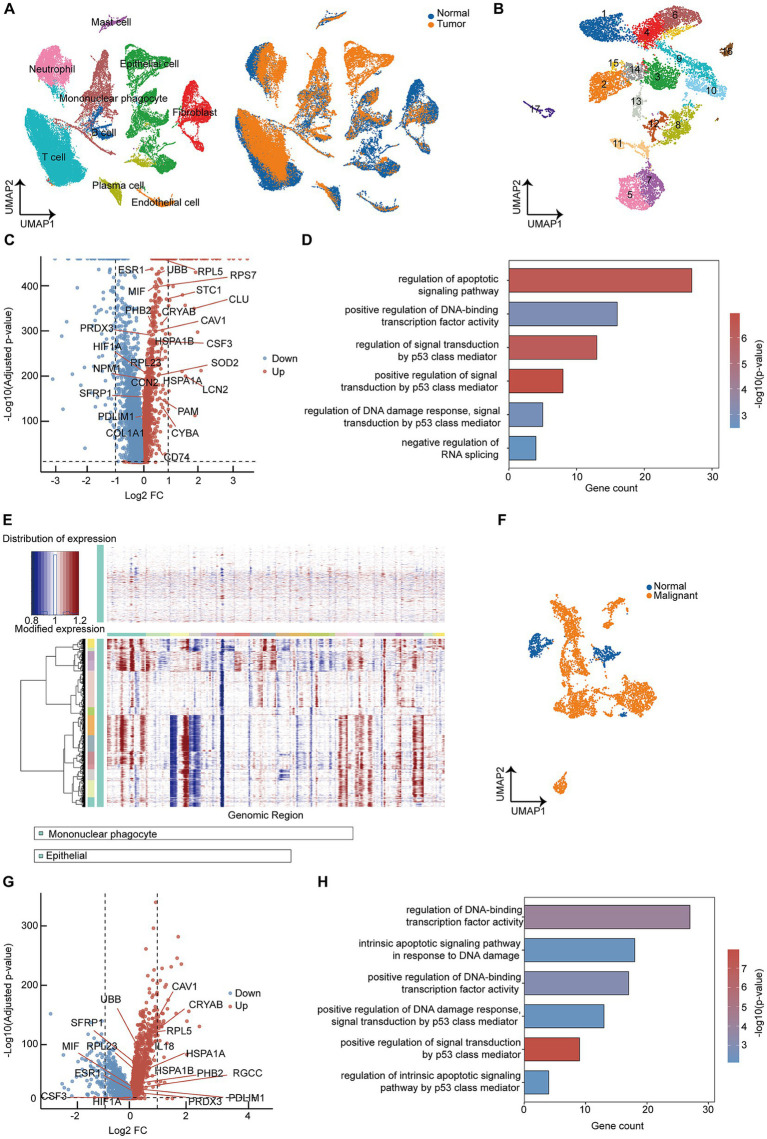
Single-cell sequencing reveals DNA damage and remodeling during cervical epithelial cell carcinogenesis. **(A)** A Uniform Manifold Approximation and Projection (UMAP) plot of the cells from patients with a normal cervix (*n =* 3), cervical cancer (*n =* 3), colored by cell types (left), and tissue groups (right). **(B)** A UMAP plot of epithelial cells from the indicated tissues. **(C)** A volcano plot showing the differentially expressed genes in epithelial cells from cervical cancer compared with the normal cervix (*p*-values calculated using two-sided Wilcoxon test). **(D)** Gene Ontology (GO) enrichment analysis of significantly upregulated genes in epithelial cells from cervical cancer. The horizontal axis (gene count) represents the number of upregulated genes associated with each term among all annotated genes in the corresponding GO category, and the terms are colored by *p*-value. **(E)** InferCNV analysis identifies malignant epithelial cells. **(F)** A UMAP plot showing malignant epithelial cells and normal epithelial cells. **(G)** A volcano plot showing differentially expressed genes between normal and malignant epithelial cells (*p*-values calculated using two-sided Wilcoxon test). **(H)** GO enrichment analysis of significantly upregulated genes in malignant epithelial cells.

To investigate the changes in epithelial cells following cervical carcinogenesis, we collected single-cell RNA sequencing data from the GEO database, including normal cervix samples (*n =* 3) and cervical cancer samples (*n =* 3) (13). Using known lineage-specific markers, we classified all cells into nine populations (Figure 1A). To detect alterations that occurred in the epithelial cells during malignant transformation, we categorized epithelial cells into 17 subclusters (Figure 1B). Subsequently, we analyzed the differentially expressed genes (DEGs) between epithelial cells derived from cervical cancer and those from normal cervical tissues. The data showed that upregulated genes in epithelial cells from tumors were primarily associated with DNA damage and aberrant activation of transcription factors (Figure 1C). This phenomenon was also supported by Gene Ontology (GO) enrichment analysis, which revealed widespread DNA damage and transcriptional dysregulation occurred in tumor-derived epithelial cells (Figure 1D).

Furthermore, we analyzed epithelial cells within tumor tissues and performed copy number variation (CNV) analysis to distinguish malignant cells from normal epithelial cells (Figures 1E,F). The upregulated genes in malignant cells were also enriched for DNA damage and transcription factor dysfunction (Figures 1G,H). Altogether, these findings suggest that epithelial cells undergo significant DNA alterations during malignant transformation in cervical cancer.

### KDM5B is upregulated in cervical cancer and is associated with poor prognosis

**Figure 2:**
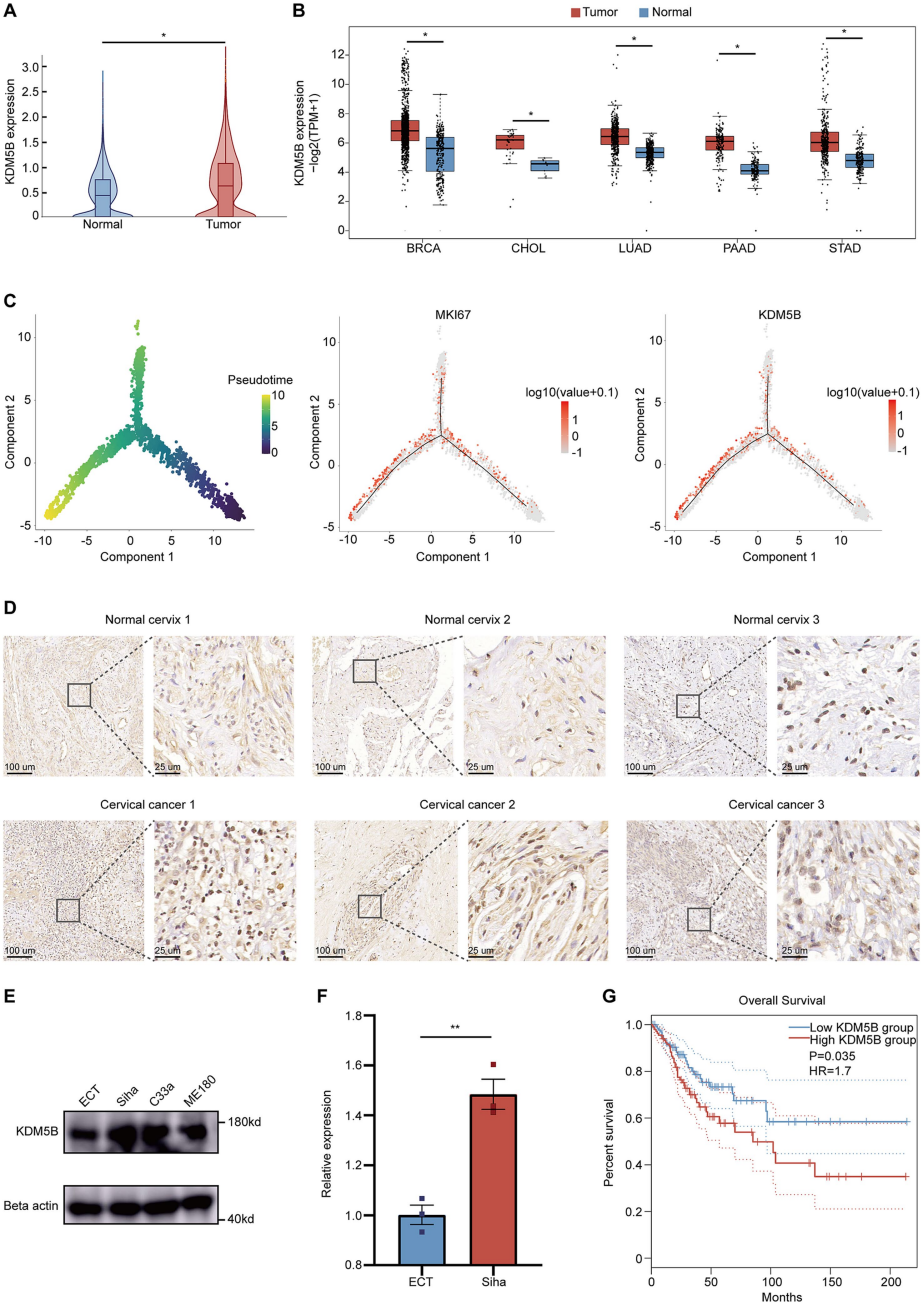
Expression of KDM5B is upregulated in cervical cancer. **(A)** Expression of KDM5B in normal cervix and cervical cancer based on scRNA-seq. The minima, maxima, center, bounds of the box and whiskers, and percentiles are indicated in the violin plots. **(B)** Expression of KDM5B across different tumors, including BRCA, CHOL, LUAD, PAAD, and STAD. **(C)** Monocle analysis for trajectory inference of epithelial cells in all samples based on scRNA-seq. Two lineages were identified. Color intensity indicates normalized gene expression. **(D)** Representative IHC staining showing KDM5B protein expression in the normal cervix and cervical cancer. Scale bars: 100 μm (left) and 25 μm (right). **(E)** Immunoblotting showing the expression of KDM5B in normal cervical cells (ECT) and cervical cancer cells (SiHa, C33a, and ME180). **(F)** qPCR showing KDM5B expression in normal cervical cells (ECT) and cervical cancer cells (SiHa) (*p*-values calculated using Student’s *t*-test). **(G)** Overall survival of patients with KDM5B_high_ and KDM5B_low_ in cervical cancer from the TCGA public database (*p* = 0.035, HR = 1.7).

We noted that the histone demethylase KDM5B is highly expressed in cervical cancer based on scRNA-Seq data (Figure 2A). Subsequently, we analyzed the bulk sequencing data of *The Cancer Genome Atlas* (TCGA) database and found that KDM5B upregulation is a common feature in multiple cancers, including breast invasive carcinoma (BRCA), cholangiocarcinoma (CHOL), lung adenocarcinoma (LUAD), pancreatic adenocarcinoma (PAAD), and stomach adenocarcinoma (STAD) (Figure 2B). KDM5B is an H3K4 demethylase critical for melanoma maintenance and drug resistance. Its regulatory role in cervical cancer was unclear. Using pseudotime analysis, we constructed the developmental trajectory of epithelial cells from tumor tissues. With reference to the malignant marker KI67, we found that KDM5B expression gradually increased during the malignant transformation process of epithelial cells (Figure 2C).

For further validation, we conducted the following experiments. First, we performed immunohistochemical staining on three normal cervical tissues and three cervical cancer tissues. As expected, KDM5B expression in epithelial cells from tumors was higher than in normal samples (Figure 2D). Second, Western blot analysis revealed that KDM5B expression level in cervical cancer cell lines, including SiHa, C33a, and ME180, was also higher compared with the normal cervical epithelial cell line (ECT; Figure 2E). Moreover, qPCR results confirmed higher KDM5B expression in SiHa cells compared with ECT (Figure 2F). Notably, this upregulated KDM5B expression was correlated with poorer patient prognosis, underscoring its crucial role in promoting the progression of cervical cancer (Figure 2G).

In summary, our results indicate that KDM5B is significantly upregulated in cervical cancer and is associated with an unfavorable prognosis.

### KDM5B promotes the malignant behaviors of cervical cancer cells

**Figure 3:**
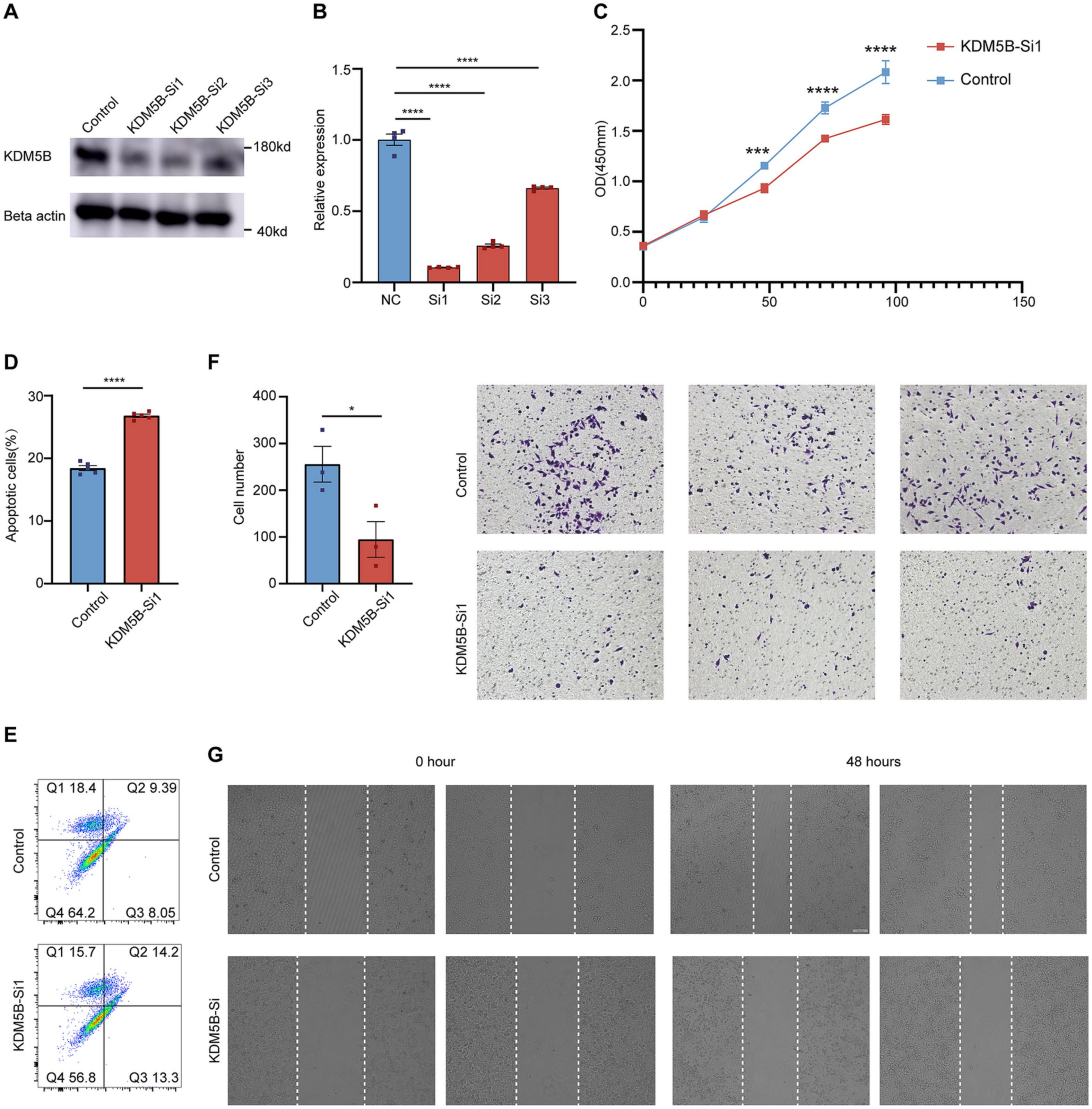
KDM5B knockdown inhibits cervical cancer progression. **(A)** Immunoblotting showing KDM5B knockdown efficiency in SiHa cells by siRNA. **(B)** qPCR showing KDM5B knockdown efficiency in SiHa cells by siRNA (*p*-values calculated using Student’s *t*-test). **(C)** Cell proliferation curve showing the effect of KDM5B on cervical cancer cells (*n =* 3 wells per group, *p*-values calculated using two-way ANOVA test). **(D)** Ratios of apoptotic cells (*n =* 5 samples per group, *p*-values calculated using Student’s *t*-test). **(E)** Representative images of flow cytometric analysis of Figure D. **(F)** Representative images of the transwell showing the KDM5B effect on tumor cell migration (*p*-values calculated using Student’s *t*-test). **(G)** Representative images of the wound healing assay showing the KDM5B effect on tumor cell migration.

To investigate the biological function of KDM5B in cervical cancer, we established KDM5B-knockdown cell lines using siRNA and confirmed knockdown efficiency by qPCR and Western blot (Figures 3A,B). We identified siRNA sequence 1 as the most effective for KDM5B knockdown and therefore selected it for all subsequent experiments.

First, CCK-8 assays revealed that KDM5B knockdown significantly suppressed the proliferation of cervical cancer cells (Figure 3C). Moreover, flow cytometric analysis with Annexin V-FITC/PI double staining showed that KDM5B silencing promoted apoptotic cell death (Figures 3D,E). Additionally, transwell migration assays and wound healing experiments indicated that the migratory ability of cancer cells was markedly impaired following KDM5B depletion (Figures 3F,G).

Together, these results suggest that KDM5B plays a critical role in promoting the malignant behaviors of cancer cells, including proliferation and migration.

### KDM5B deficiency induces the IFN response in cancer cells

**Figure 4:**
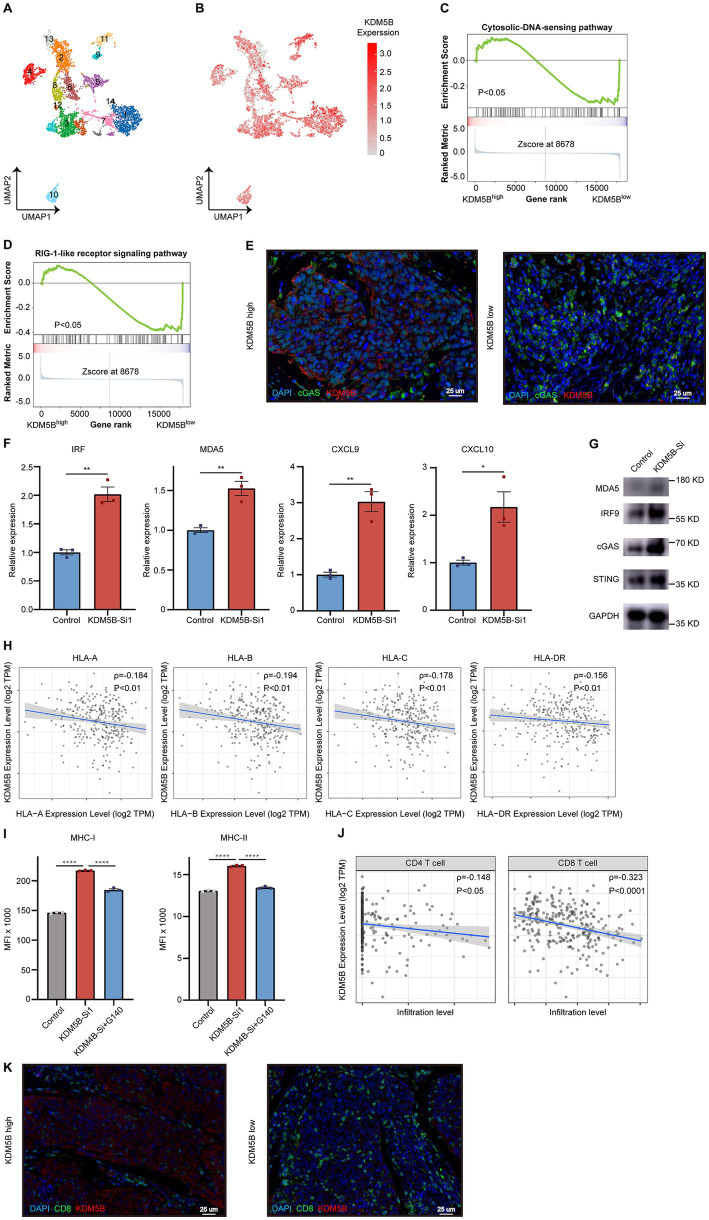
KDM5B inhibits the activation of the IFN-*γ* pathway in cervical cancer. **(A)** A UMAP plot of epithelial cells from cervical cancer tissues. **(B)** A UMAP plot showing the expression of KDM5B among epithelial subclusters of cervical cancer. **(C)** GSEA enrichment for signatures linked to the cytosolic DNA sensing signaling pathway of KDM5B^low^ epithelial cells compared with KDM5B^high^ epithelial cells. **(D)** GSEA enrichment for signatures linked to the RIG-1-like receptor signaling pathway of KDM5B^low^ epithelial cells compared with KDM5B^high^ epithelial cells. **(E)** Immunofluorescence of human CC tissues showing the spatial relationship between cGAS expression and IGSF3 expression (*n =* 6 samples). **(F)** qPCR showing the expression of genes related to IFN response activation in SiHa cells after KDM5B knock down (*p*-values calculated using Student’s *t*-test). **(G)** Representative images showing the expression of interferon-stimulated genes (ISGs). **(H)** Scatter plots showing an inverse Spearman’s correlation between KDM5B expression and antigen-presenting molecule expression. Data are shown as a fitted line with 95% confidence level and were analyzed by a linear model. **(I)** Representative histogram and bar graph of mean fluorescence intensity (MFI) showing an increase in △MFI of antigen-presenting molecule expression after KDM5B knockdown or treatment with G140 in comparison with normal controls (*n =* 3 per group, *p*-values calculated using Student’s *t*-test). **(J)** Scatter plots showing an inverse Spearman’s correlation between KDM5B expression and CD4^+^/CD8^+^ T cells infiltration. Data are shown as a fitted line with 95% confidence level and were analyzed using a linear model. **(K)** Immunofluorescence of human CC tissues showing the spatial relationship between CD8^+^ T cell infiltration and epithelial cells with high (left) versus low (right) IGSF3 expression (*n =* 6 samples).

To further detect the consequences of KDM5B loss in cancer cells, we subclustered the epithelial cells in cervical cancer into two types based on the KDM5B expression (Figures 4A,B). We observed that KDM5B was highly expressed in the Epi_8, Epi_2, and Epi_4 subpopulations but exhibited low expression in the Epi_11, Epi_12, and Epi_13 subpopulations. We further analyzed the differentially expressed genes between these subpopulations and found that pathways associated with IFN response—such as the RIG-I-like receptor signaling pathway and the cytosolic DNA-sensing pathway—were significantly enriched in KDM5B-low cells (Figures 4C,D) (14, 15). Subsequently, the results of immunofluorescence have suggested that KDM5B expression is negatively correlated with cGAS, a key molecule in DNA stress (Figure 4E). Then, we performed qPCR on *KDM5B^KD^* and *KDM5B^NC^* SiHa cells and found that molecules involved in interferon-stimulated genes (ISGs), including MDA5, IRF9, CXCL9, and CXCL10, were markedly elevated under KDM5B deficiency (Figure 4F). Consistently, the protein levels of MDA5, IRF9, and cGAS were also increased (Figure 4G). These results indicate that KDM5B deficiency promotes the IFN response in cervical cancer cells.

Notably, the IFN-*γ* pathway not only promotes the malignant biological behaviors of cancer cells but is also positively associated with tumor antigen presentation, suggesting that KDM5B contributes to immune escape (16, 17). Gene correlation analysis showed a significant negative relationship between KDM5B and key antigen presentation-related genes (Figure 4H). In addition, we observed upregulated expression of MHC class I and II molecules in tumor cells following KDM5B knockdown (Figure 4I). To further confirm that the enhanced antigen presentation capacity upon KDM5B loss is dependent on the induction of the IFN pathway, we treated *KDM5B^KD^* SiHa cells with G140, a DNA sensor inhibitor that suppresses ISG expression. We noted that the upregulation of both MHC-I and MHC-II was effectively reversed by G140 (Figure 4I). Together, these results demonstrate that KDM5B promotes malignant behaviors in cancer cells and attenuates antigen presentation capability by suppressing IFN response.

Therefore, we hypothesized that targeting KDM5B could potentially enhance the function of both CD4^+^ and CD8^+^ T cells, which was further supported by immune cell infiltration analysis using the CIBERSORT algorithm (Figure 4J). We then performed immunofluorescence and noted that more CD8^+^ T cells infiltrated the KDM5B low-expression areas (Figure 4K).

### KDM5B recruits FOXG1 to suppress gene expression

**Figure 5:**
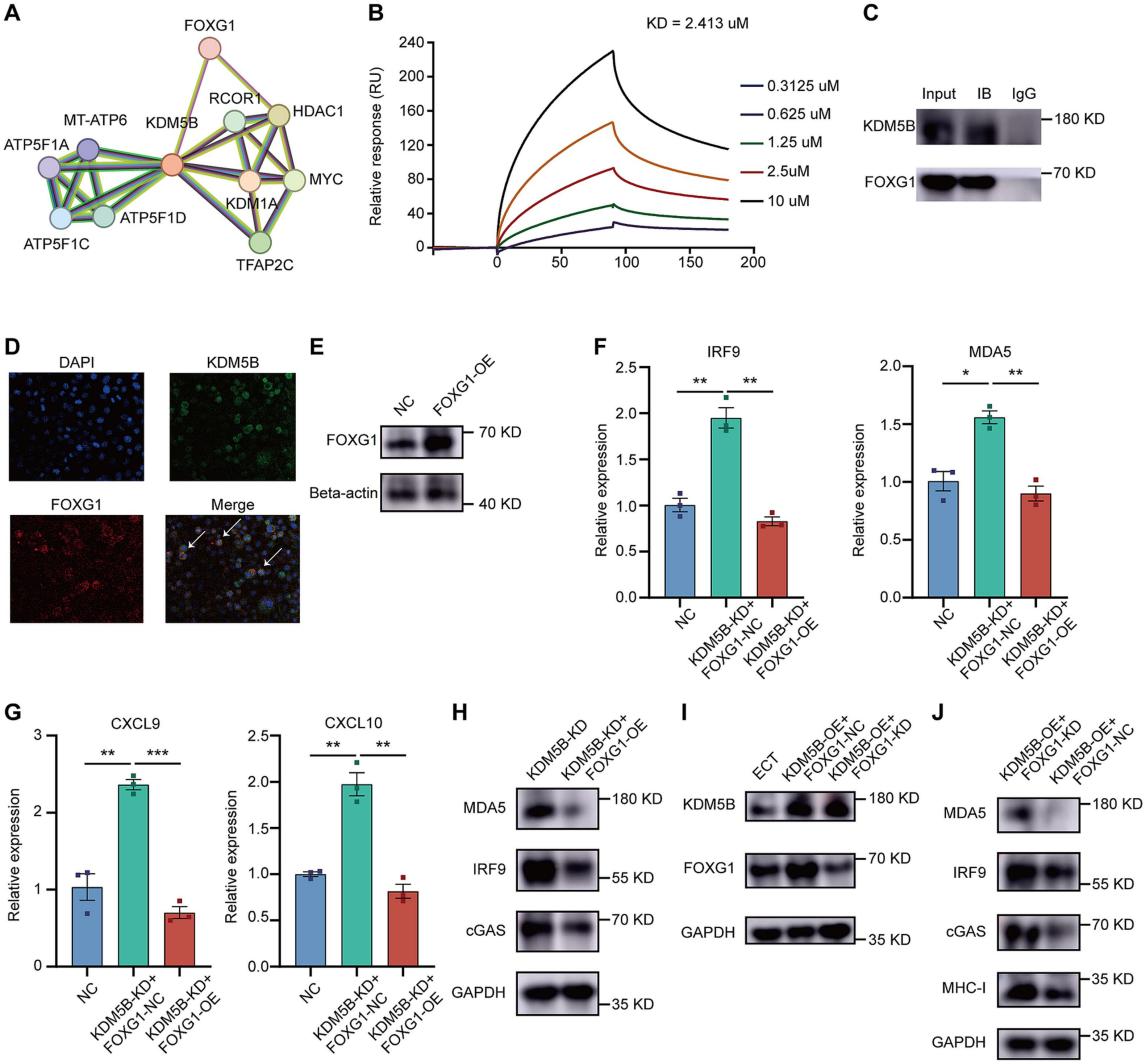
KDM5B recruits FOXG1 to suppress gene expression. **(A)** The PPI network showing potential binding proteins of KDM5B. **(B)** SPR sensorgrams of the interaction between KDM5B and FOXG1. **(C)** Representative images from the Co-IP experiment with KDM5B and FOXG1. **(D)** Microscopic views from the IF experiment showing the co-localization of KDM5B and FOXG1. Scale bar represents 4 μm. **(E)** Representative images of FOXG1 expression in KDM5B^KD^ SiHa cells and control. **(F,G)** qPCR showing the expression of genes related to ISGs in KDM5B^KD^ SiHa cells after FOXG1 overexpression (*p*-values calculated using Student’s *t*-test). **(H)** Representative images of ISG expression after FOXG1 overexpression. **(I)** Representative images of KDM5B and FOXG1 expression in different cell lines. **(J)** Representative images of ISG and MHC-I expression in FOXG1^KD^ KDM5B^OE^ SiHa cells.

To investigate how KDM5B influences IFN responses, we searched the STRING public database and explored proteins that may interact with KDM5B. We noted that the transcriptional repressor FOXG1 potentially interacts with KDM5B (Figure 5A). Therefore, we hypothesized that KDM5B might recruit FOXG1 to suppress ISG expression. To validate such a hypothesis, we performed the following experiments. First, surface plasmon resonance (SPR) demonstrated that KDM5B binds robustly to FOXG1, with an affinity of 2.413 μM (Figure 5B). Next, co-immunoprecipitation (Co-IP) experiments confirmed the endogenous direct interaction between KDM5B and FOXG1 in cancer cells (Figure 5C). Furthermore, immunofluorescence analysis revealed overlapping fluorescence signals of KDM5B and FOXG1 in the nuclei of tumor cells, providing additional evidence for their endogenous interaction (Figure 5D). Moreover, in *KDM5B^KD^* cervical cancer cells, overexpression of FOXG1 counteracted the activation of IFN responses caused by KDM5B deficiency (Figures 5E–H). These results support that FOXG1 mediates the transcriptional repression function of KDM5B.

To further confirm this hypothesis, we conducted a series of rescue experiments. When we knocked down FOXG1 in KDM5B overexpression SiHa cells, the IFN pathway remained activated (Figures 5I–J). The antigen presentation capacity was enhanced (Figure 5J). Altogether, our experimental results demonstrate that KDM5B interacts with the repressive transcription factor FOXG1 to suppress the IFN response, thereby inhibiting the expression of related genes.

### KDM5B is a potential immunotherapy target in cervical cancer

**Figure 6:**
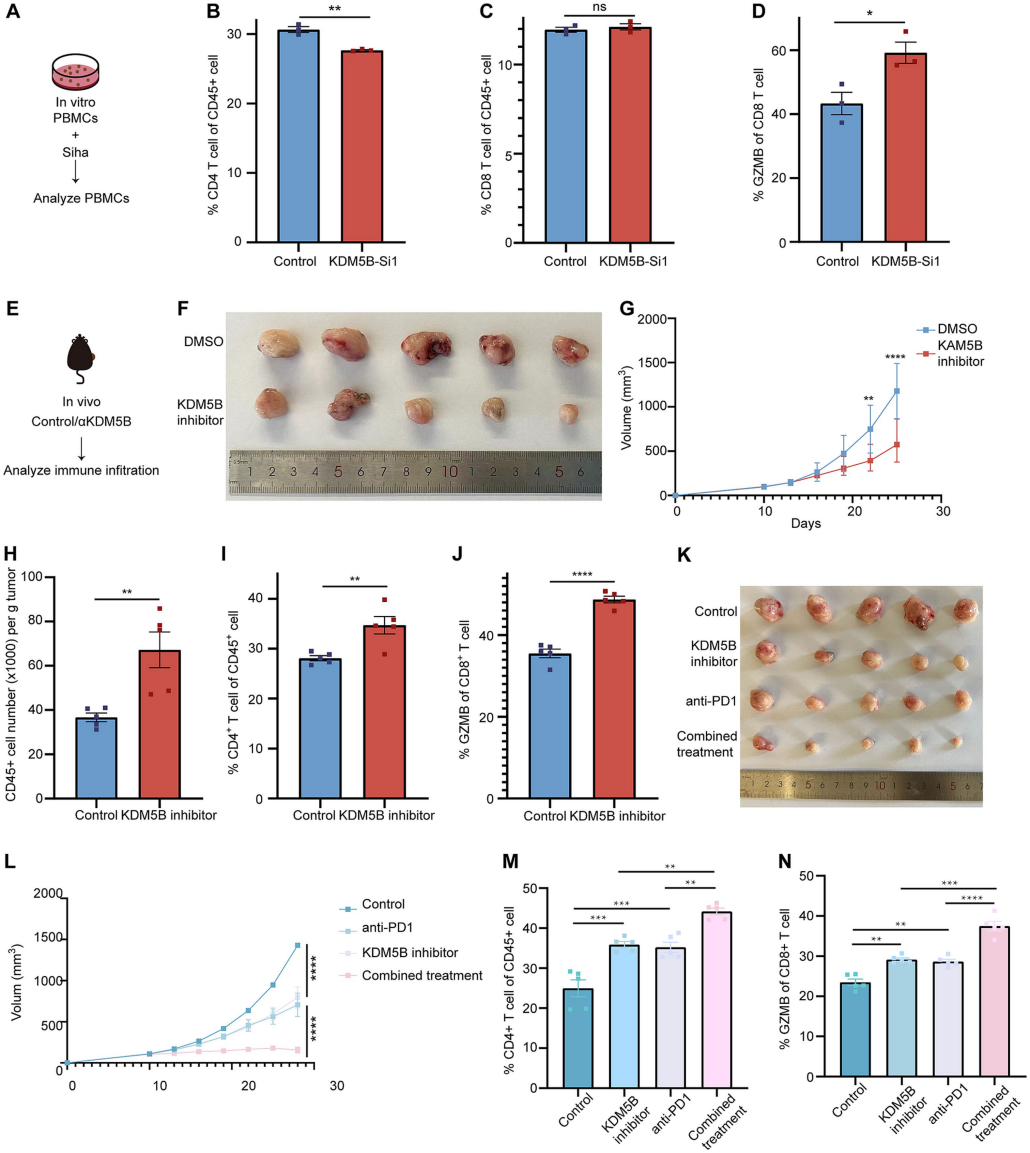
KDM5B is a potential therapeutic target in cervical cancer. **(A)** Experimental design for Figures 5B–D. **(B)** Ratios of CD4^+^ T cells among CD45^+^ cells in the *KDM5B^NC^*/*KDM5B^KD^* SiHa cells and PBMC coculture system (*n =* 3 samples per group, *p*-values calculated using paired *t*-test). **(C)** Ratios of CD8^+^ T cells among CD45^+^ cells in the *KDM5B^NC^*/*KDM5B^KD^* SiHa cells and PBMC coculture system (*n =* 3 samples per group, *p*-values calculated using paired *t*-test). **(D)** Ratios of GZMB expression on CD8^+^ T cells in the *KDM5B^NC^*/*KDM5B^KD^* SiHa cells and PBMC coculture system (*n =* 3 samples per group, *p*-values calculated using paired *t*-test). **(E)** Experimental design for Figures 5F–G. **(F)** Representative gross morphology of tumor tissues from mice-bearing TC1 cells treated with dimethyl sulfoxide (DMSO)/KDM5B inhibitor. **(G)** Tumor growth curve showing the effect of KDM5B inhibitor on tumor growth (*n =* 5 mice per group, *p*-values calculated using two-way ANOVA test). **(H)** Numbers of CD45^+^ cells per gram of tumors in mice-bearing TC1 cells treated with DMSO or KDM5B inhibitor (*n =* 5 mice per group, *p*-values calculated using Student’s *t*-test). **(I)** Ratios of CD4^+^ T cells among CD45^+^ cells in tumors of mice-bearing TC1 cells treated with DMSO or KDM5B inhibitor (*n =* 5 mice per group, *p*-values calculated using Student’s *t*-test). **(J)** Ratios of GZMB expression on CD8^+^ T cells in tumors of mice-bearing TC1 cells treated with DMSO or KDM5B inhibitor (*n =* 5 mice per group, *p*-values calculated using Student’s *t*-test). **(K)** Representative gross morphology of tumor tissues from mice bearing TC1 cells that received different treatments. **(L)** Tumor growth curve of mice bearing TC1 cells received different treatments (*n =* 5 mice per group, *p*-values calculated using two-way ANOVA test). **(M)** Ratios of CD4^+^ T cells among CD45^+^ cells in tumors of mice-bearing TC1 cells received different treatments (*n =* 5 mice per group, *p*-values calculated using Student’s *t*-test). **(N)** Ratios of GZMB expression on CD8^+^ T cells in tumors of mice-bearing TC1 cells received different treatments (*n =* 5 mice per group, *p*-values calculated using Student’s *t*-test).

To investigate the impact of KDM5B on the tumor immune microenvironment in cervical cancer, we first co-cultured *KDM5B^NC^* or *KDM5B^KD^* SiHa cells with peripheral blood mononuclear cells (PBMCs) and assessed the changes in immune cell populations (Figure 6A). We observed a significant increase in the proportion of CD4^+^ T cells upon KDM5B deficiency (Figures 6B,C); while KDM5B knockdown did not alter the fraction of CD8^+^ T cells, it significantly enhanced their cytotoxic activity (Figure 6D), suggesting that KDM5B loss potentiates antitumor immunity.

To further detect the potential of KDM5B as an immunotherapy target, we administered the KDM5B inhibitor (KDM5B-IN-4) to mice bearing subcutaneous tumors at a dose of 30 mg/kg/day (Figure 6E). Compared with the control group, targeting KDM5B produced a significant antitumor effect (Figures 6F,G). Flow cytometry analysis further revealed a marked increase in CD45^+^ immune cell infiltration (Figure 6H), elevated proportions of CD4^+^ T cells (Figure 6I), and enhanced cytotoxic activity in CD8^+^ T cells in the treatment group (Figure 6J).

Given that KDM5B deficiency enhances antigen presentation and promotes T cell recognition, we next explored the *in vivo* effect of targeting KDM5B in combination with anti-PD1. Mice bearing TC1 cells were randomly assigned to four groups: control, KDM5B inhibitor, anti-PD1, and KDM5B inhibitor combined with anti-PD1. As expected, the combination treatment group exhibited the most potent therapeutic efficacy (Figure 6K). Tumor growth was significantly suppressed (Figure 6L), accompanied by a marked increase in CD4^+^ T cell infiltration and enhanced cytotoxic activity of CD8^+^ T cells (Figures 6M,N).

In summary, our results demonstrate that KDM5B is a potential therapeutic target in cervical cancer and has an additive effect to anti-PD1 treatment.

## Discussion

Cervical cancer is one of the most common malignancies among women. With the diversification of cancer treatment modalities, therapeutic strategies for cervical cancer have been continuously refined, resulting in significant benefits to patients (18). However, the clinical outcomes of these treatments remain limited, primarily due to the incomplete understanding of the biological mechanisms underlying the malignant transformation of cervical epithelial cells. In this study, we discovered that DNA damage and epigenetic remodeling occurred in cervical cancer cells. Notably, KDM5B, a well-known histone-modifying enzyme, was significantly upregulated. KDM5B markedly promoted the malignant behaviors of cancer cells, including proliferation and migration. On the other hand, KDM5B deficiency enhanced tumor antigen presentation capacity in cervical cancer. We also found that KDM5B exerts its regulatory function by recruiting FOXG1 to suppress the IFN response. Through *in vitro* drug experiments, our results suggest that KDM5B is a promising therapeutic target for cervical cancer, both as a single agent or in combination with anti-PD1.

Genomic instability is one of the most prevalent hallmarks of human cancer and plays a critical role in tumorigenesis (5). It is typically characterized by defects in DNA damage repair mechanisms, chromosomal structural and numerical abnormalities, and microsatellite instability (19–21). Single-cell sequencing studies have revealed a significant accumulation of DNA damage during the malignant transformation of cervical epithelial cells (13, 22). Concurrently, the dysfunction of key tumor suppressor genes such as p53 further impairs cell cycle control and DNA repair capacity, thereby promoting the development of a malignant phenotype (23, 24). This intrinsic genetic instability not only accelerates the accumulation of mutations in oncogenes and tumor suppressor genes but also provides a genetic basis for tumor heterogeneity, driving clonal evolution and adaptation to the tumor microenvironment, ultimately leading to tumor progression.

KDM5B, a key histone-modifying enzyme, regulates gene expression through epigenetic mechanisms. Notably, previous studies have primarily focused on the mechanism by which the KDM family regulates gene expression through histone modification (10). However, our research reveals that KDM5B exerts its function by recruiting the inhibitory transcription factor FOXG1 to suppress the interferon (IFN) response in tumor cells. When we treated *KDM5B^KD^* SiHa cells with G140, a specific cGAS inhibitor (25), the expression levels of MHC-I and MHC-II were rescued, suggesting that the KAM5B–FOXG1 axis inhibits antigen presentation by suppressing the IFN response. KDMs usually bind to cofactors or interact with transcription factors that can enhance their activity, targeting, and function (26). In our study, we propose that the interaction between KDM5B and FOXG1 likely enhances the accessibility of FOXG1 to target gene promoter regions and stabilizes its binding to DNA. Given that the IFN pathway positively regulates MHC expression (17), our findings that KDM5B deficiency promotes the expression of both MHC-I and MHC-II, thereby enhancing antitumor immunity, are consistent with this mechanism.

HPV infection is a major cause of cervical cancer. E7 from HPV can bind to inactivated STING and inhibit the IFN response (27, 28). Moreover, E6 and E7 promote KDM expression to drive epigenetic reprogramming and further inhibit the downstream pathway (29). Therefore, targeting KDM5B represents a promising therapeutic strategy. It may not only reverse HPV-induced epigenetic reprogramming in infected epithelial cells but also concurrently activate STING and disrupt its interaction with E7. This dual effect would effectively rescue the host IFN response, thereby mounting a robust defense against HPV infection. More importantly, KDM5B-IN-4 is a specific KDM5B inhibitor with excellent selectivity and demonstrates strong antitumor effects, whether serving as a single agent or in combination with anti-PD1 therapy (30). The relationship between the KDM5B-FOXG1 and the IFN response warrants further investigation in future studies.

This study confirms the fundamental role of DNA damage accumulation and tumor suppressor gene inactivation in driving the development of cervical cancer. Furthermore, we reveal that the crucial function of the histone-modifying enzyme KDM5B in promoting tumor proliferation, migration, and immune evasion is mediated by its recruitment of the transcriptional repressor FOXG1. Future investigations should continue to uncover novel regulatory mechanisms within the KDM family. More importantly, targeting KDM5B significantly enhances immune infiltration and activates antitumor T-cell responses, highlighting its potential as a promising therapeutic target for cervical cancer immunotherapy. Considerable effort should be devoted to developing optimized therapeutic strategies that combine KDM5B inhibition with existing immunotherapies.

## Data Availability

Publicly available datasets were analyzed in this study. This data can be found at: dataset (GSE120575) in GEO public database.
