# Supplementary figures and images for: KDM5B recruits FOXG1 to suppress the IFN response, leading to malignant progression and immune evasion in cervical cancer
Source: Front Med (Lausanne). 2026 Apr 1;13:1749536. doi: 10.3389/fmed.2026.1749536 (PMC13079605; doi:10.3389/fmed.2026.1749536)

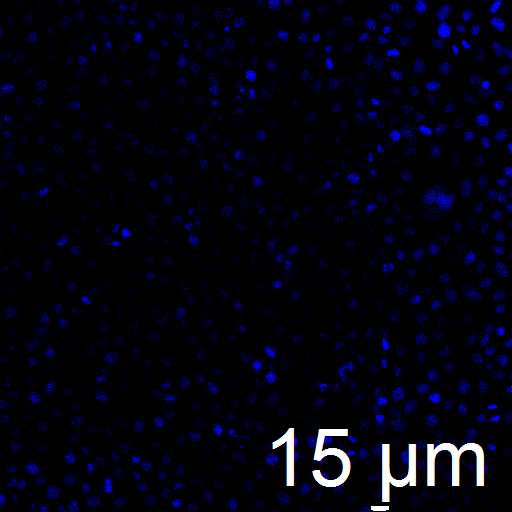

Supplement: Supplementary file 1 [file Data_Sheet_1.zip › DAPI.tif]

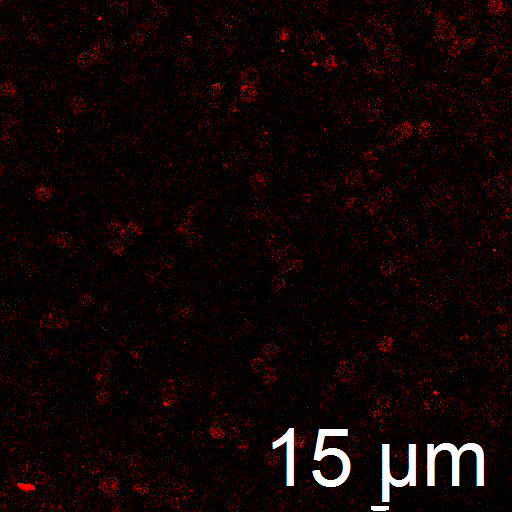

Supplement: Supplementary file 1 [file Data_Sheet_1.zip › FOXG1.tif]

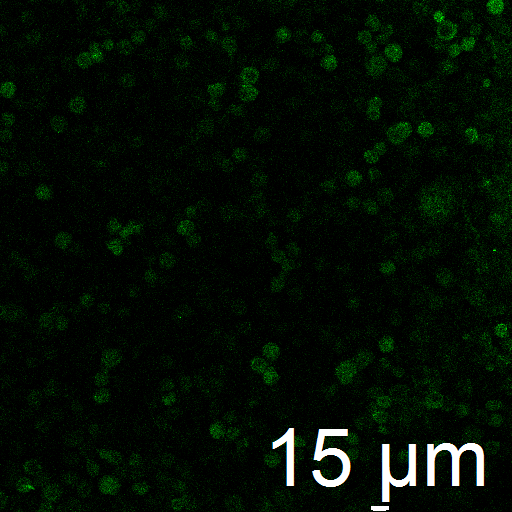

Supplement: Supplementary file 1 [file Data_Sheet_1.zip › KDM5B.tif]

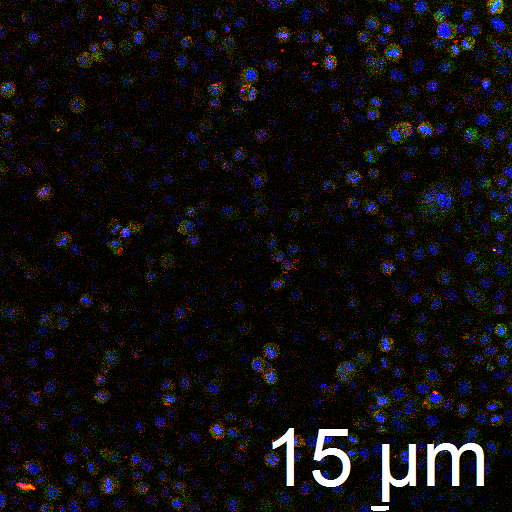

Supplement: Supplementary file 1 [file Data_Sheet_1.zip › Merge.tif]

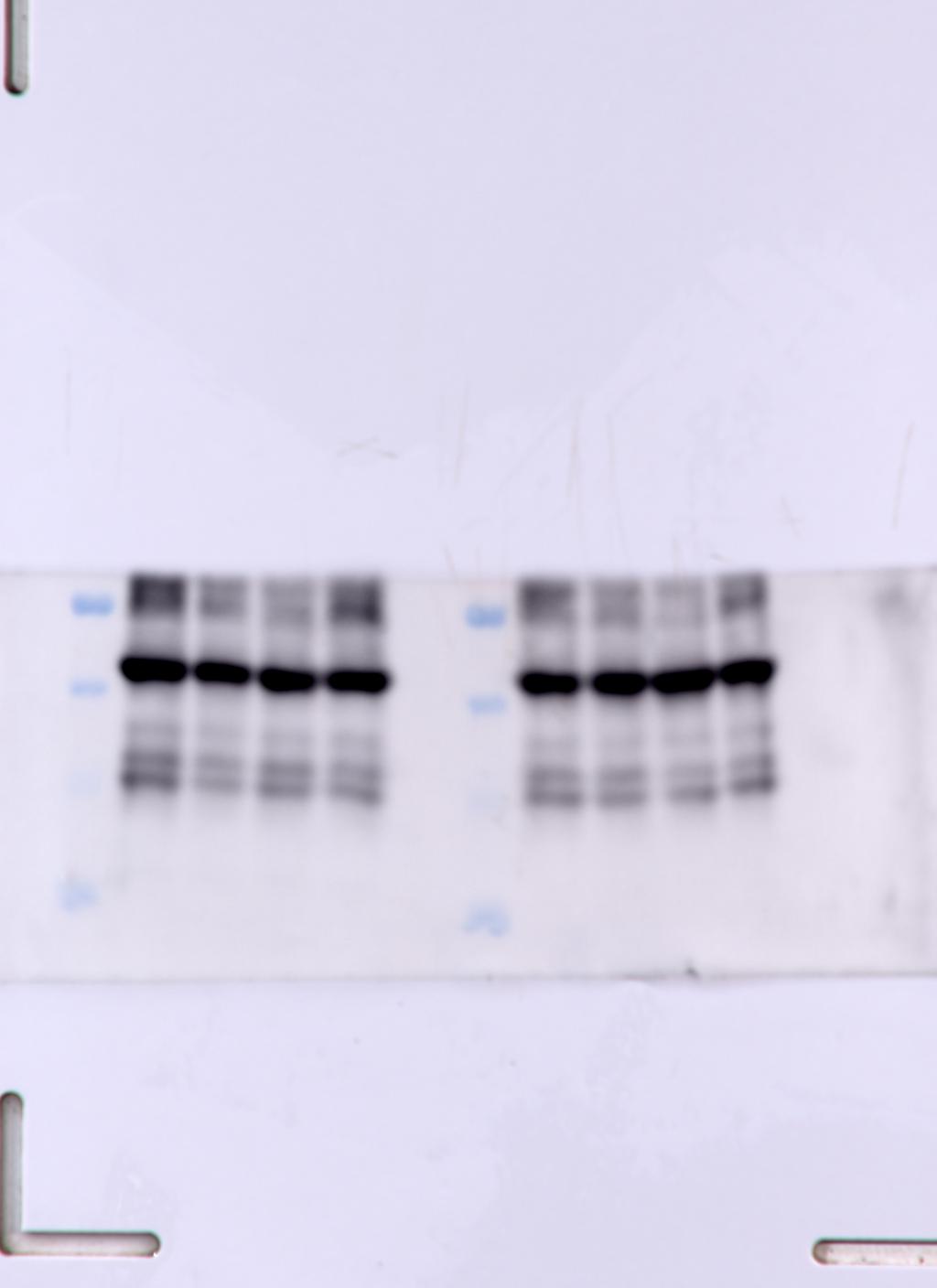

Supplement: Supplementary file 2 [file Data_Sheet_2.zip › Figure 2/beta-actin.jpg]

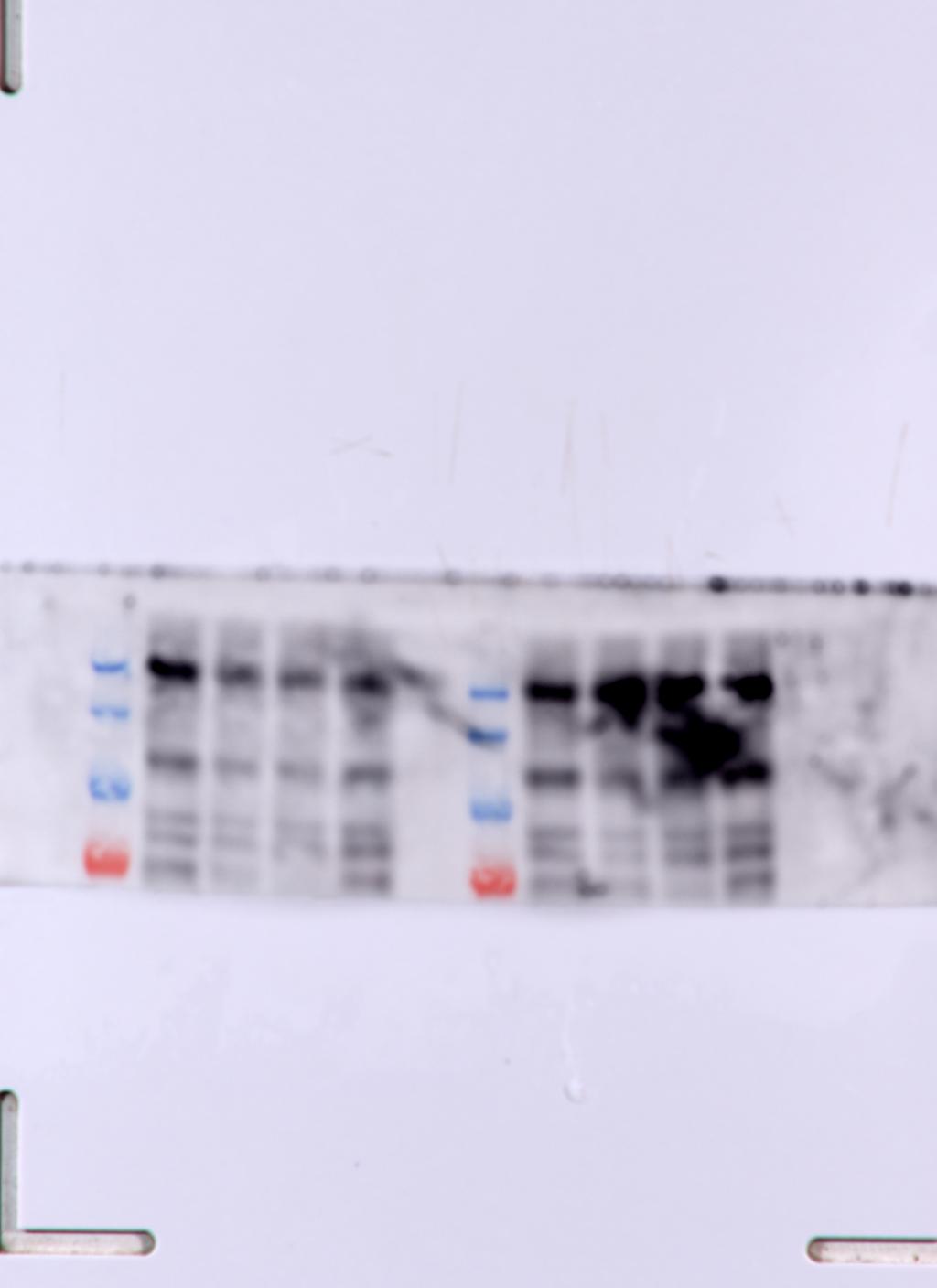

Supplement: Supplementary file 2 [file Data_Sheet_2.zip › Figure 2/KDM5B.jpg]

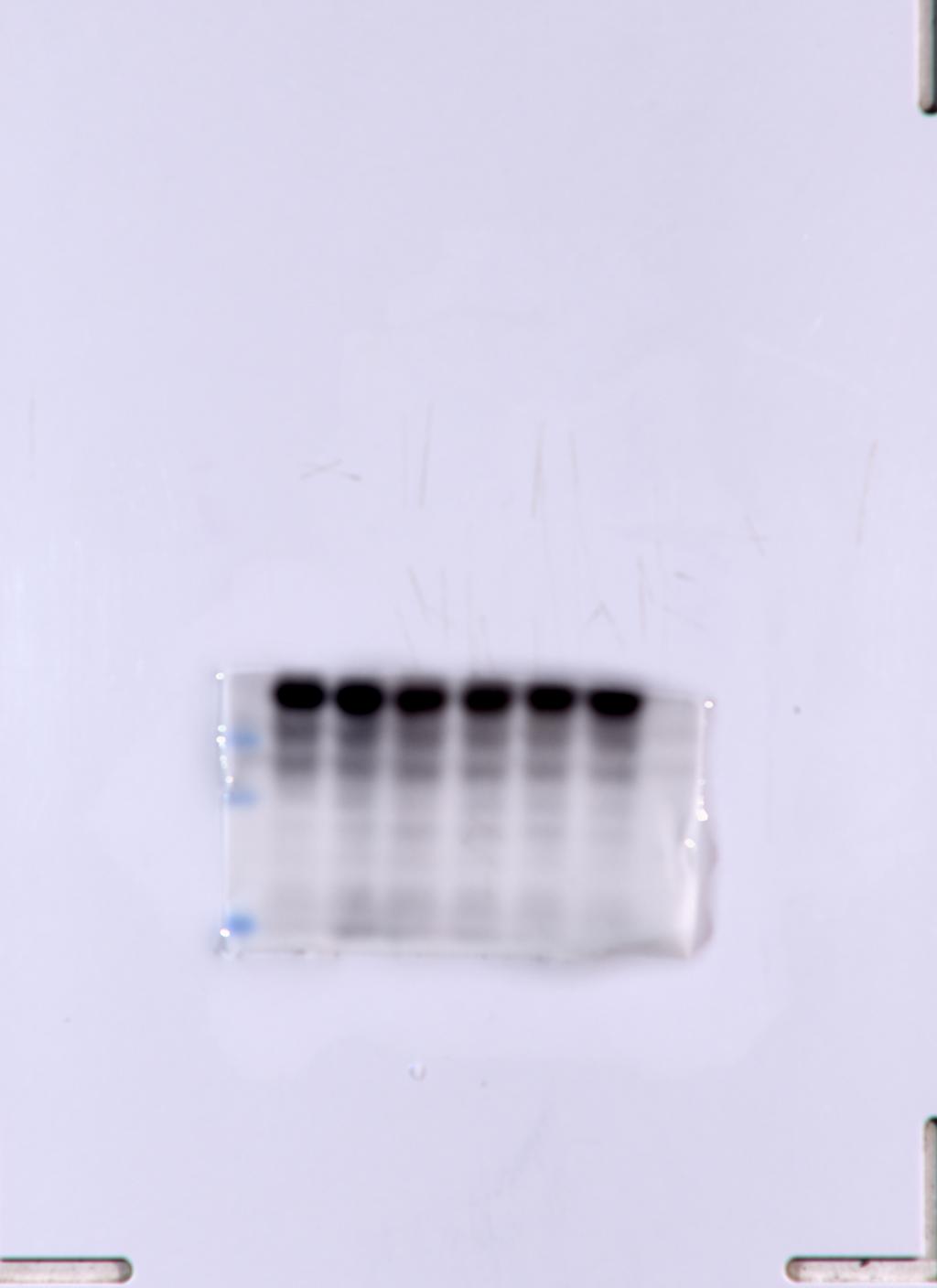

Supplement: Supplementary file 2 [file Data_Sheet_2.zip › Figure 4/GAPDH.jpg]

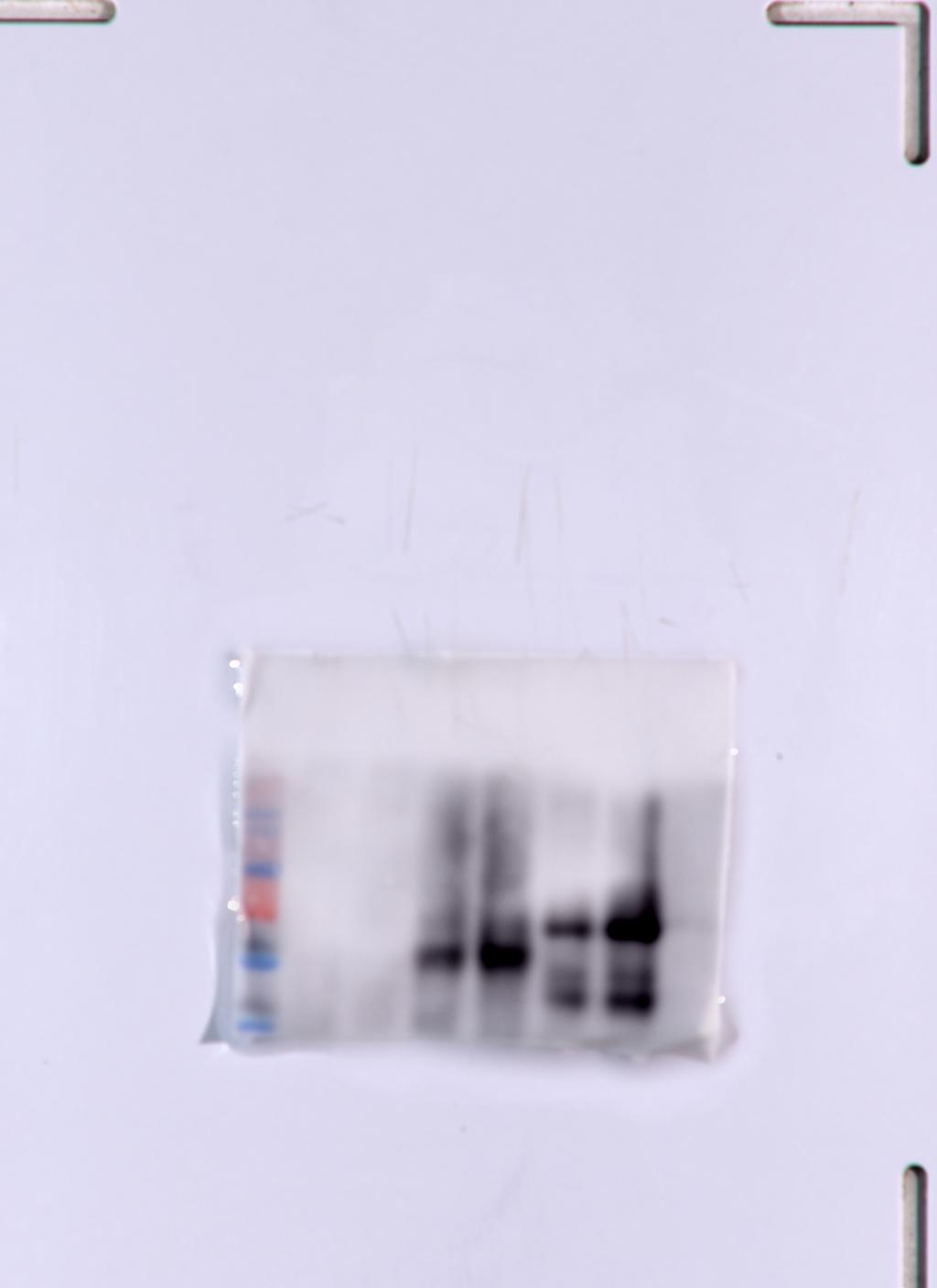

Supplement: Supplementary file 2 [file Data_Sheet_2.zip › Figure 4/IRF9 and cGAS.jpg]

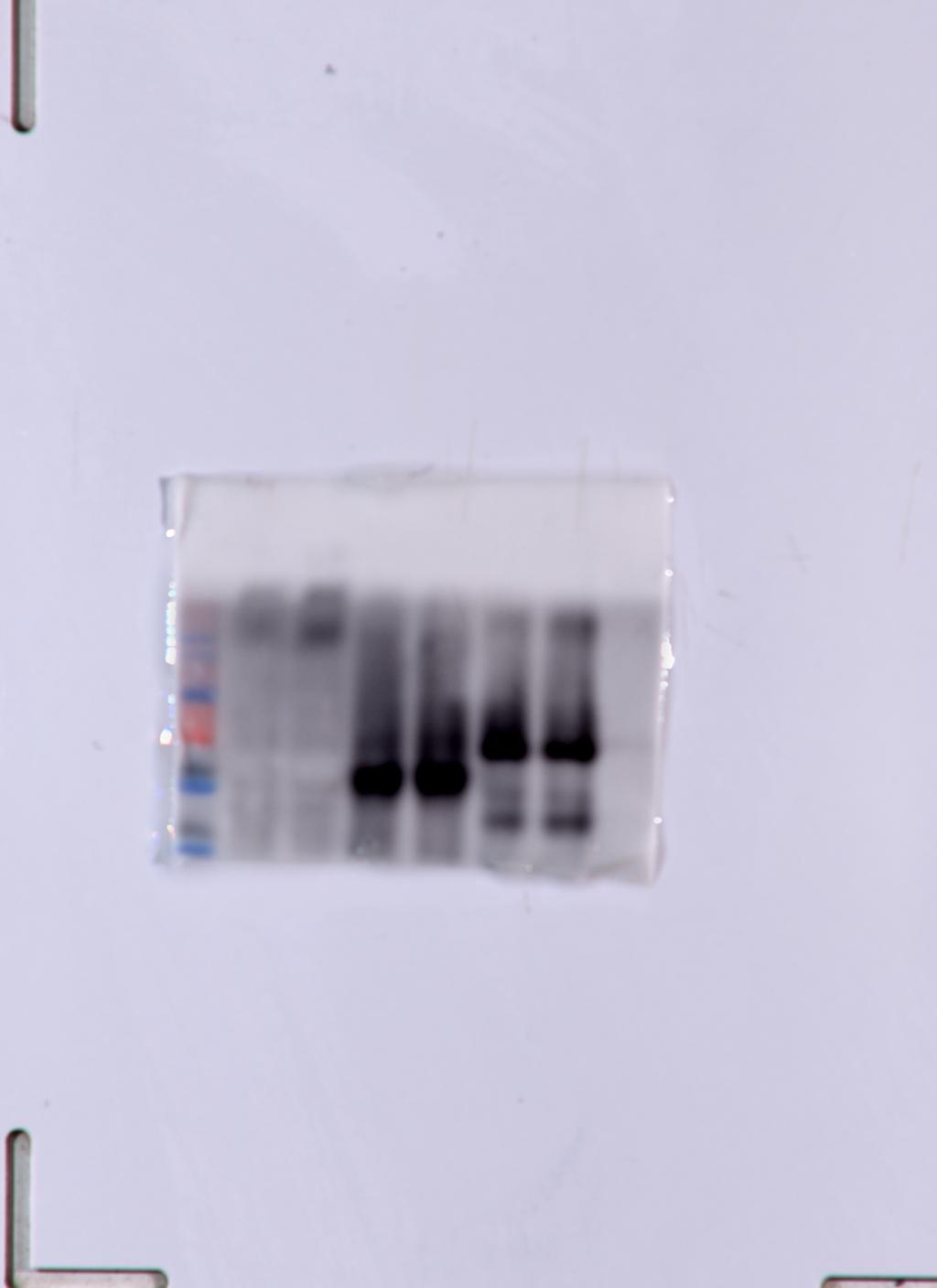

Supplement: Supplementary file 2 [file Data_Sheet_2.zip › Figure 4/MDA5.jpg]

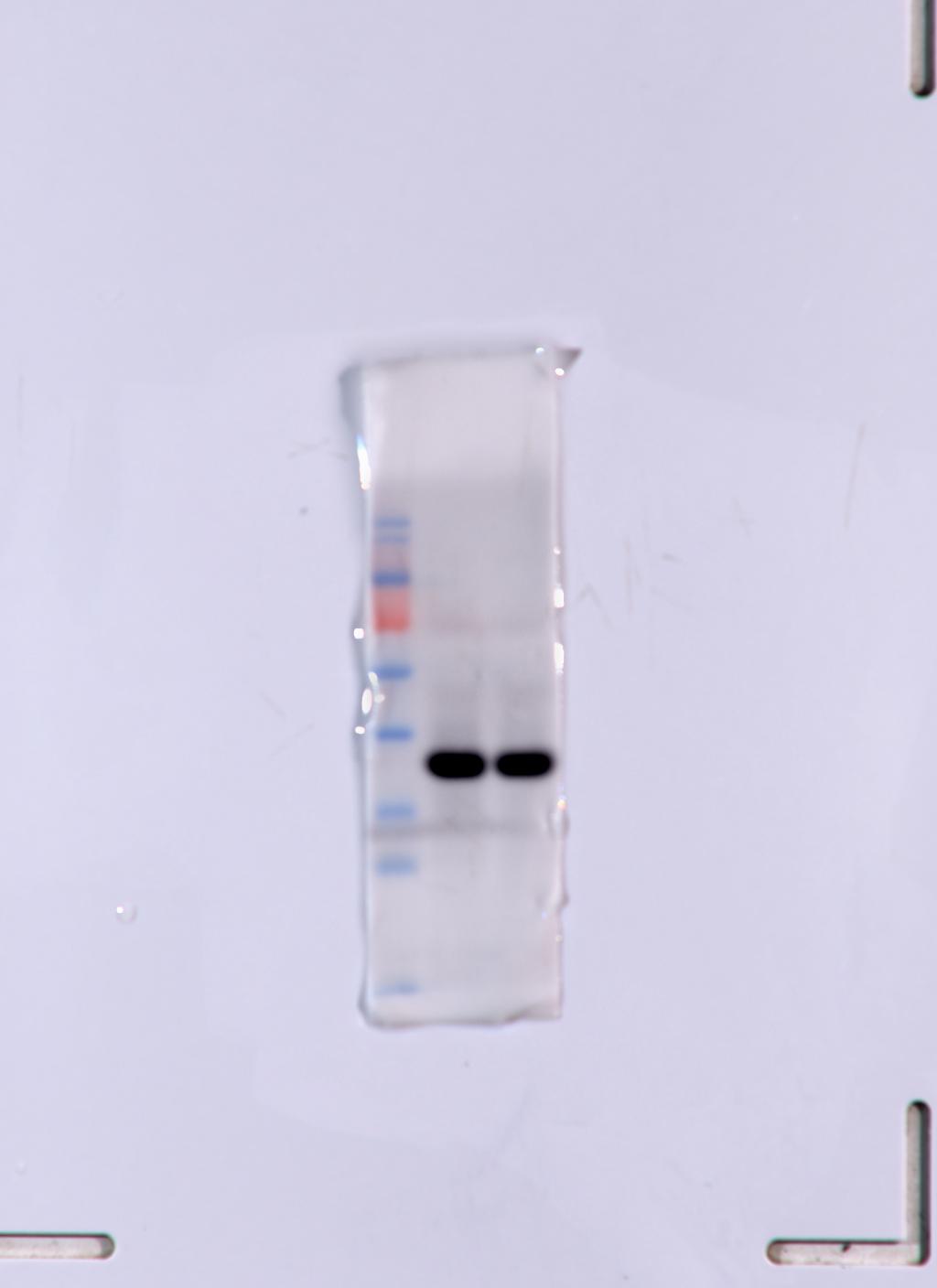

Supplement: Supplementary file 2 [file Data_Sheet_2.zip › Figure 4/Sting-GAPDH.jpg]

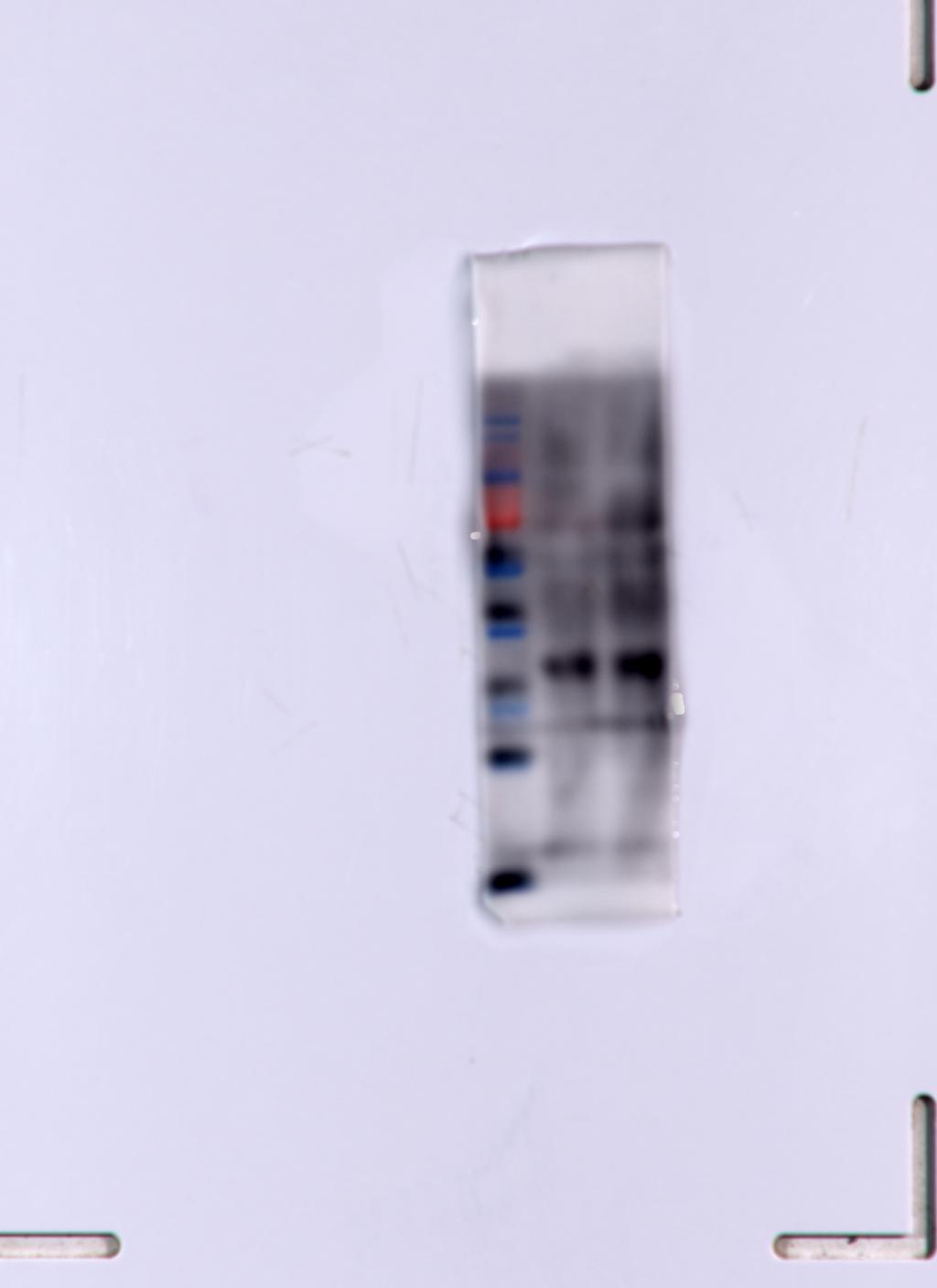

Supplement: Supplementary file 2 [file Data_Sheet_2.zip › Figure 4/Sting.jpg]

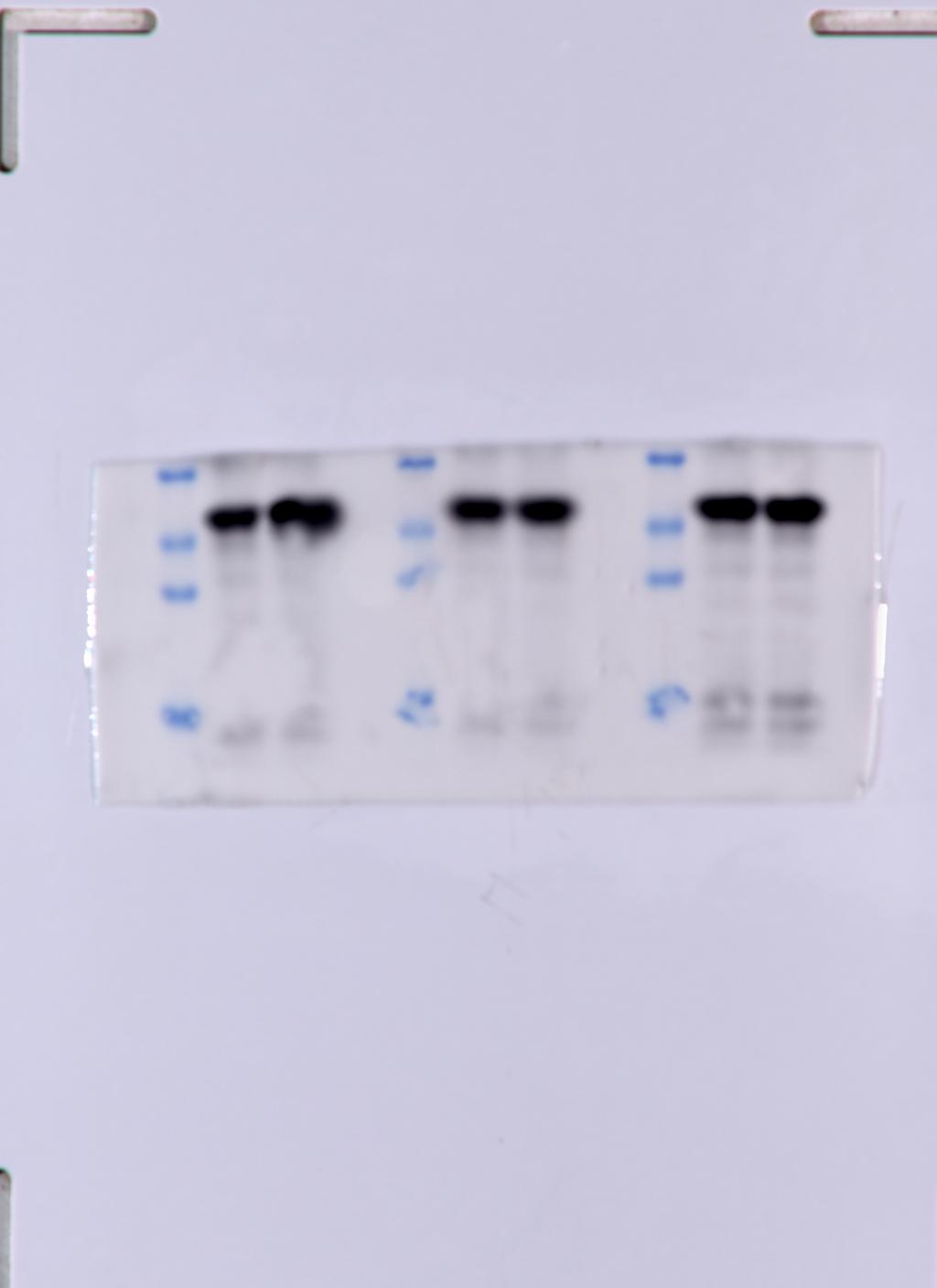

Supplement: Supplementary file 2 [file Data_Sheet_2.zip › Figure 5/cGAS-GAPDH.jpg]

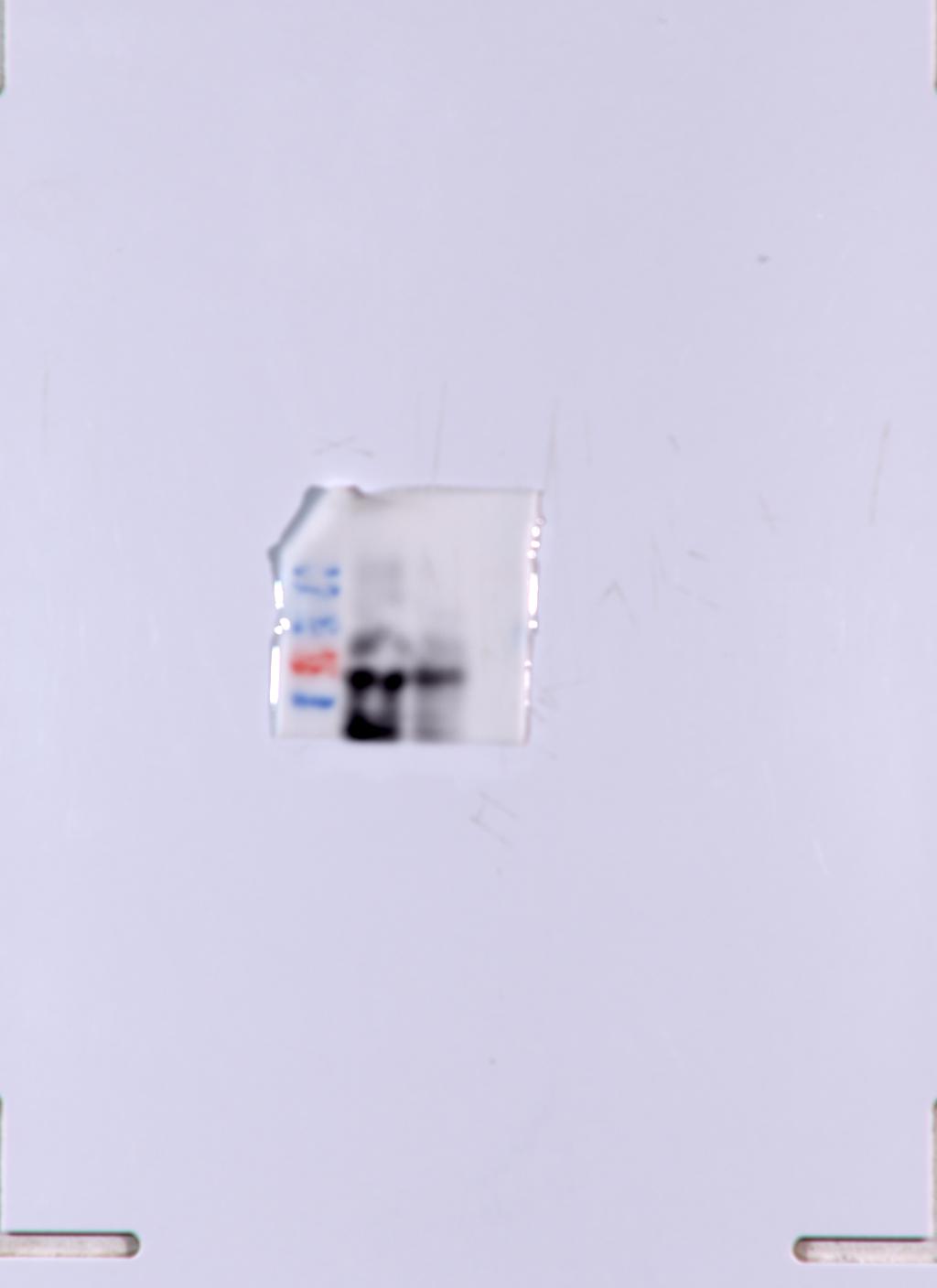

Supplement: Supplementary file 2 [file Data_Sheet_2.zip › Figure 5/cGAS.jpg]

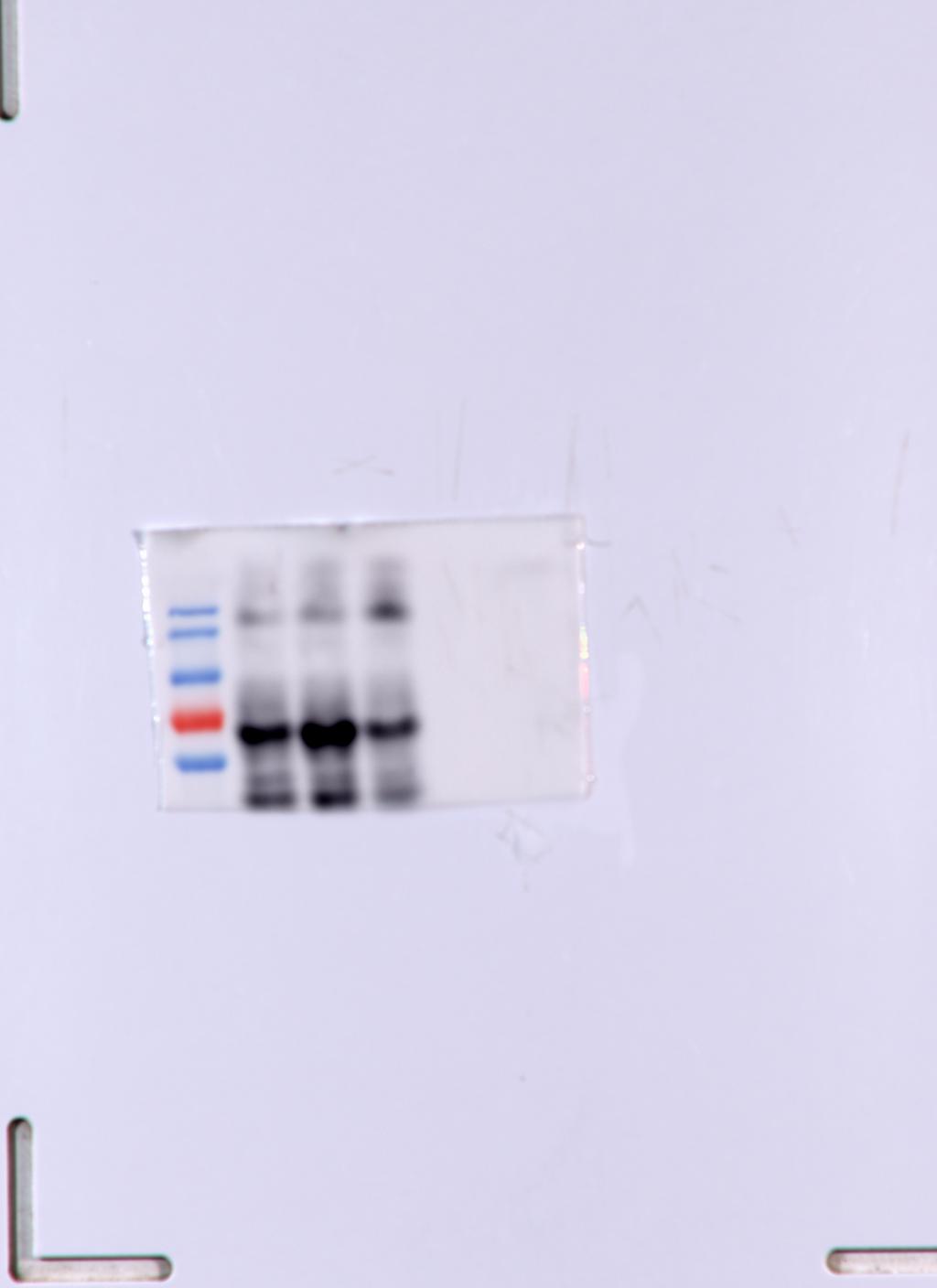

Supplement: Supplementary file 2 [file Data_Sheet_2.zip › Figure 5/FOXG1.jpg]

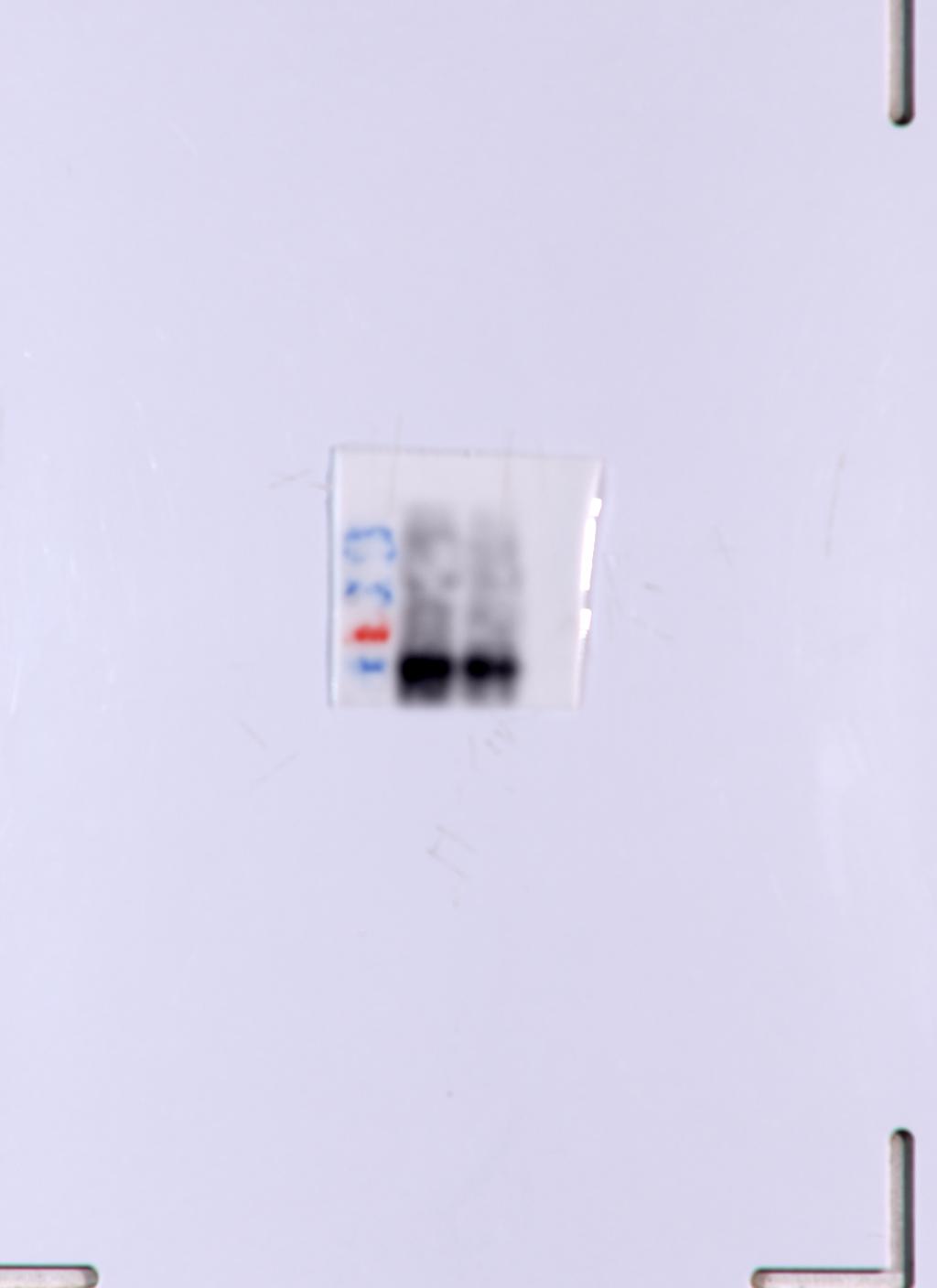

Supplement: Supplementary file 2 [file Data_Sheet_2.zip › Figure 5/IRF9.jpg]

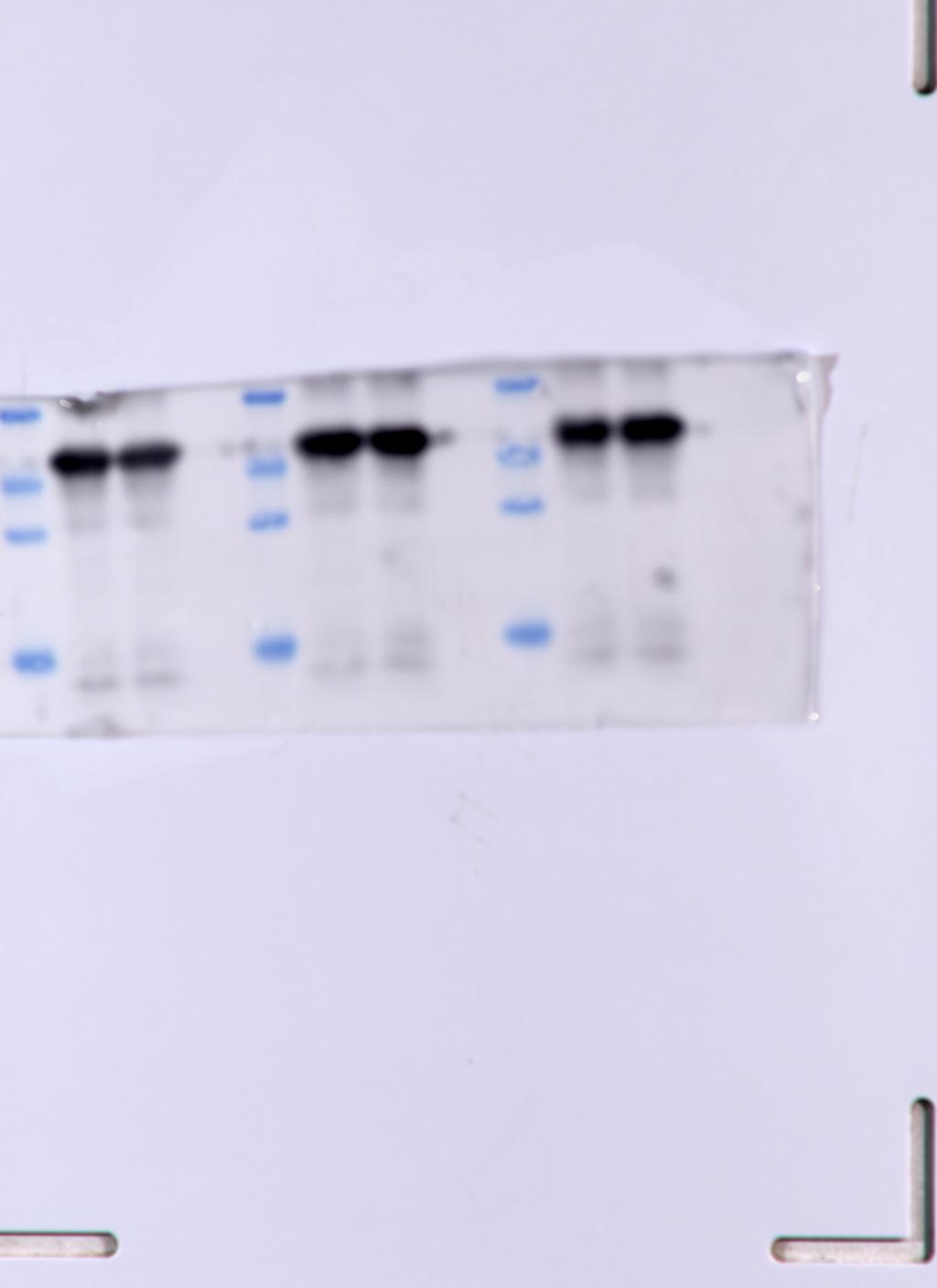

Supplement: Supplementary file 2 [file Data_Sheet_2.zip › Figure 5/ISG-GAPDH.jpg]

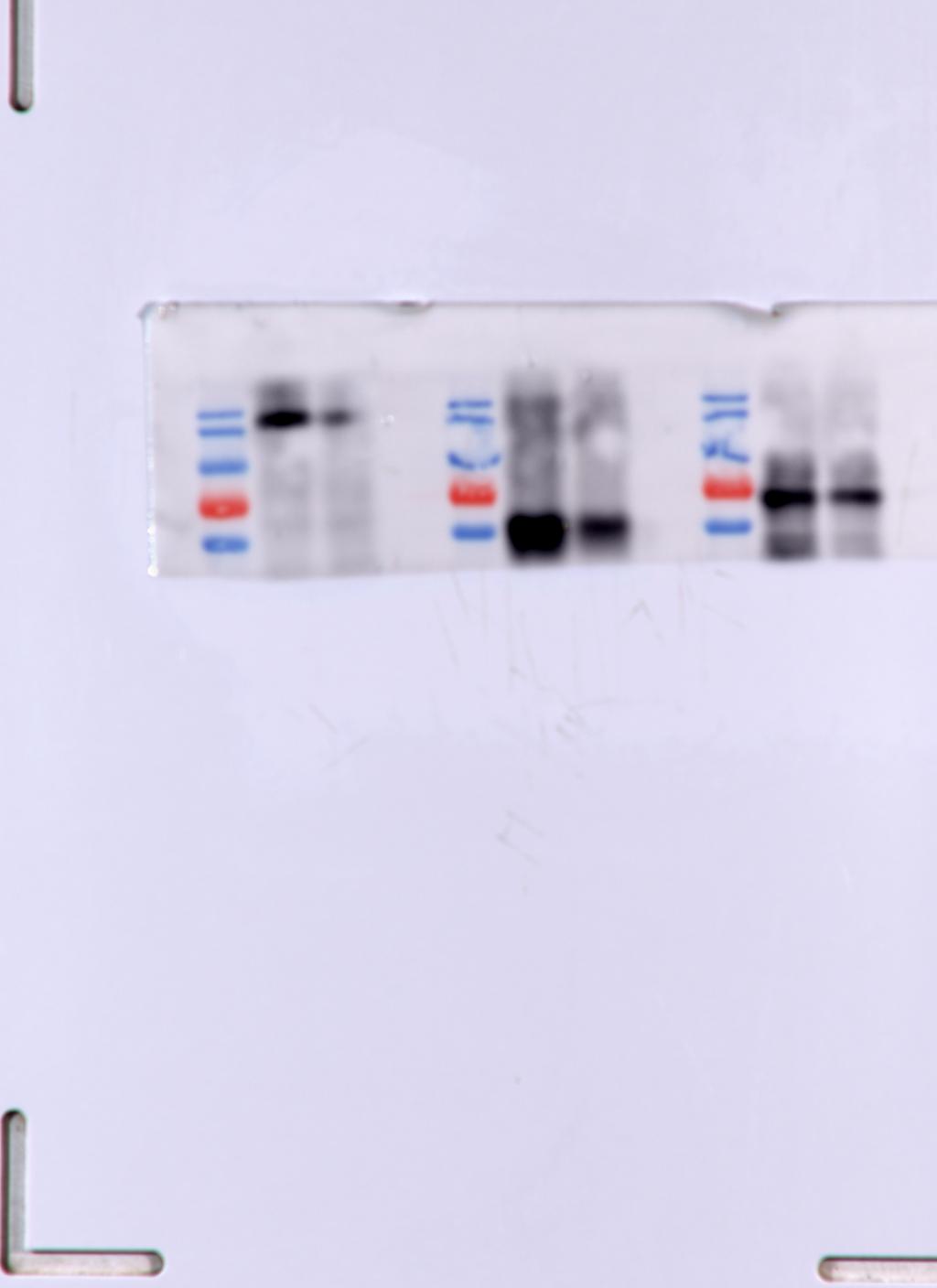

Supplement: Supplementary file 2 [file Data_Sheet_2.zip › Figure 5/ISG.jpg]

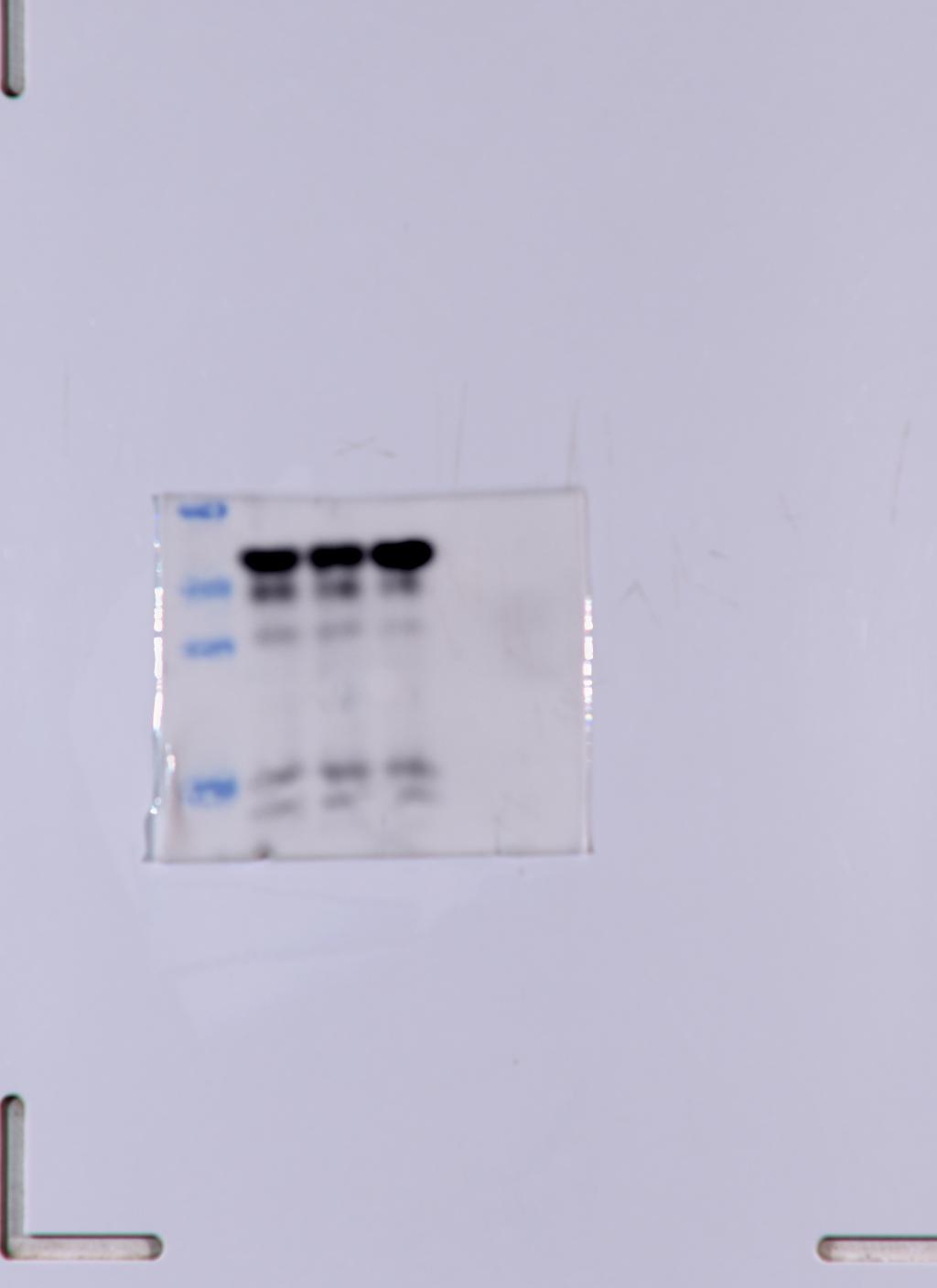

Supplement: Supplementary file 2 [file Data_Sheet_2.zip › Figure 5/KDM5B-FOXG1-GAPDH.jpg]

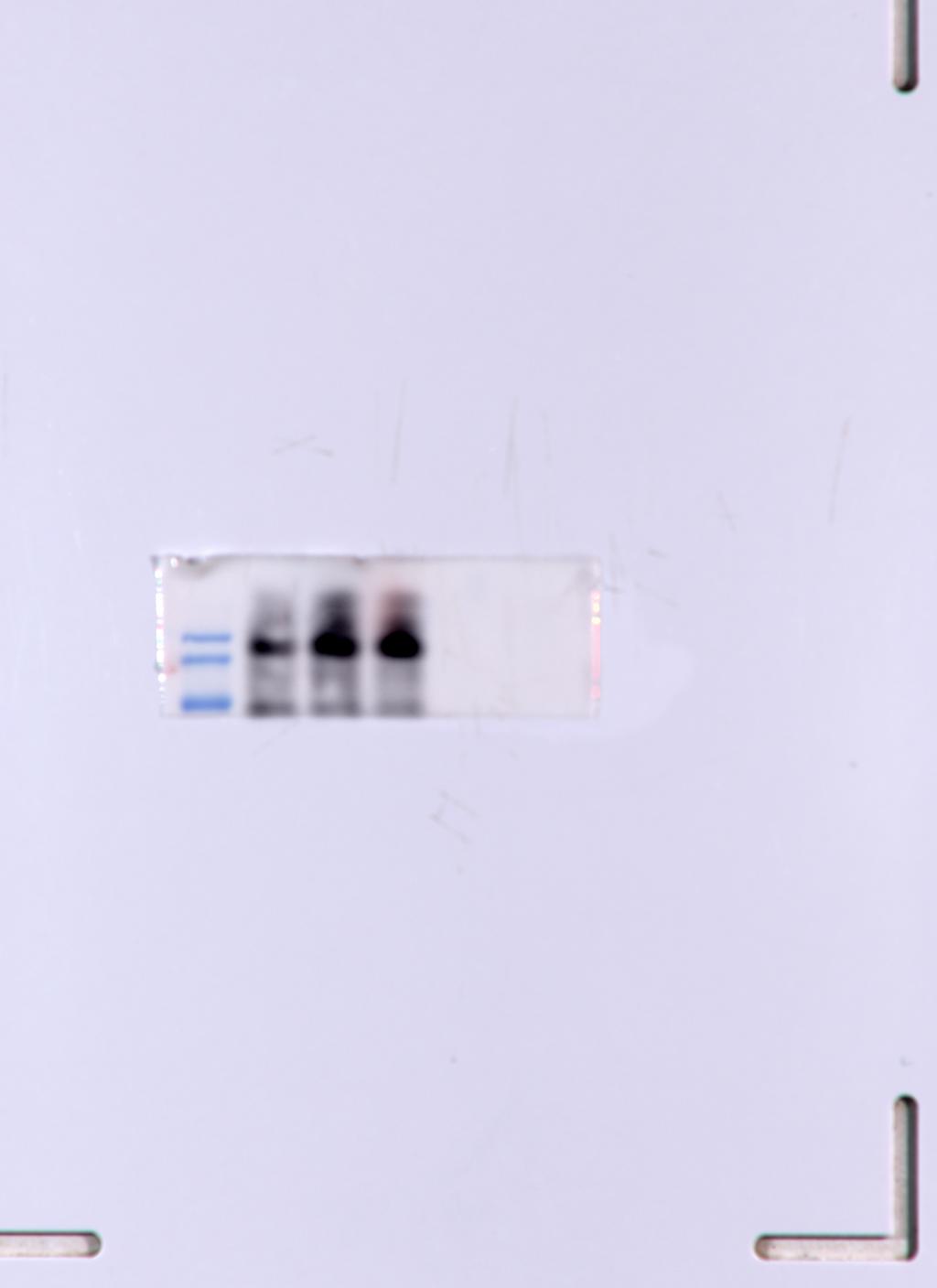

Supplement: Supplementary file 2 [file Data_Sheet_2.zip › Figure 5/KDM5B.jpg]

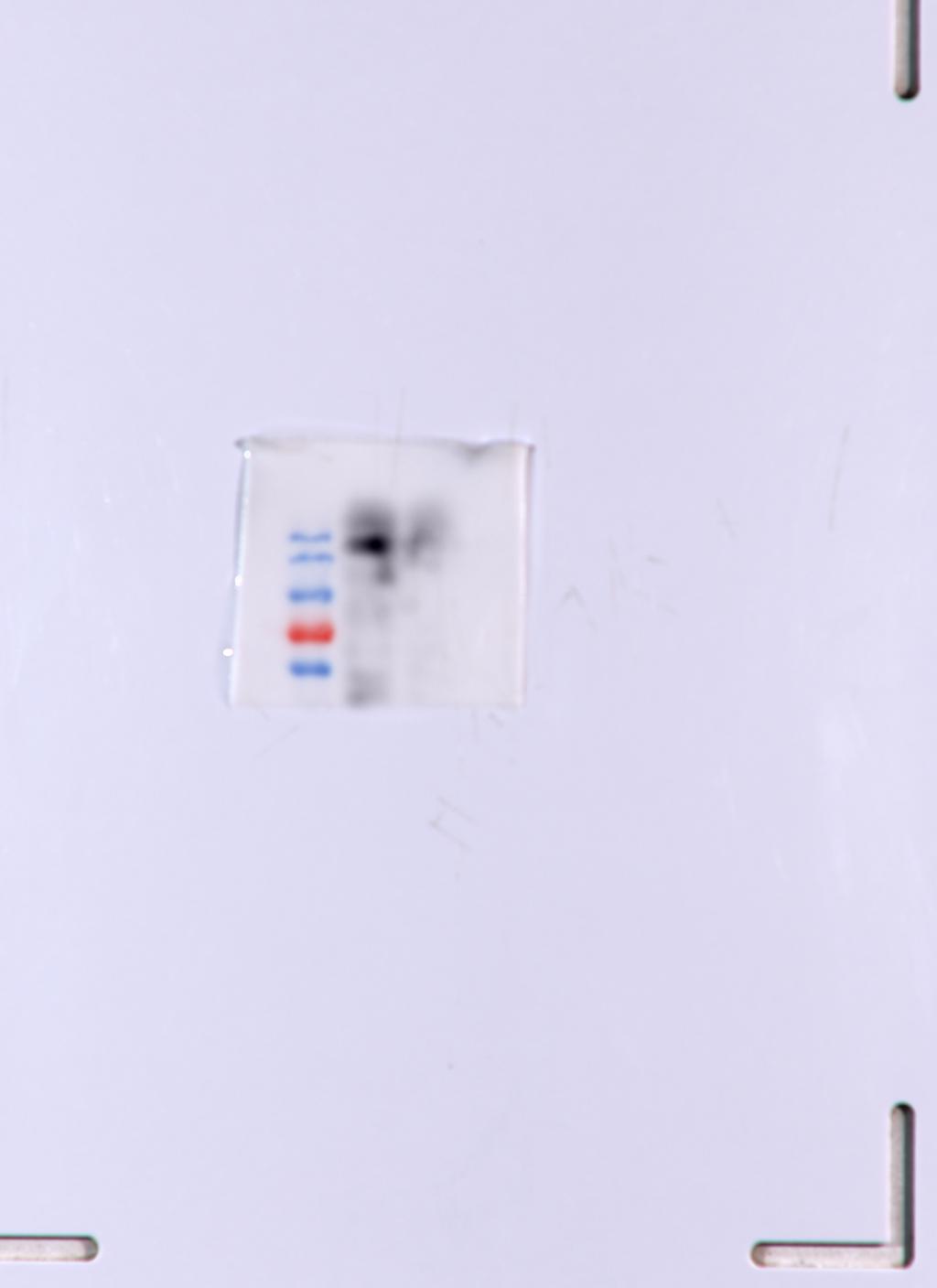

Supplement: Supplementary file 2 [file Data_Sheet_2.zip › Figure 5/MDA5.jpg]

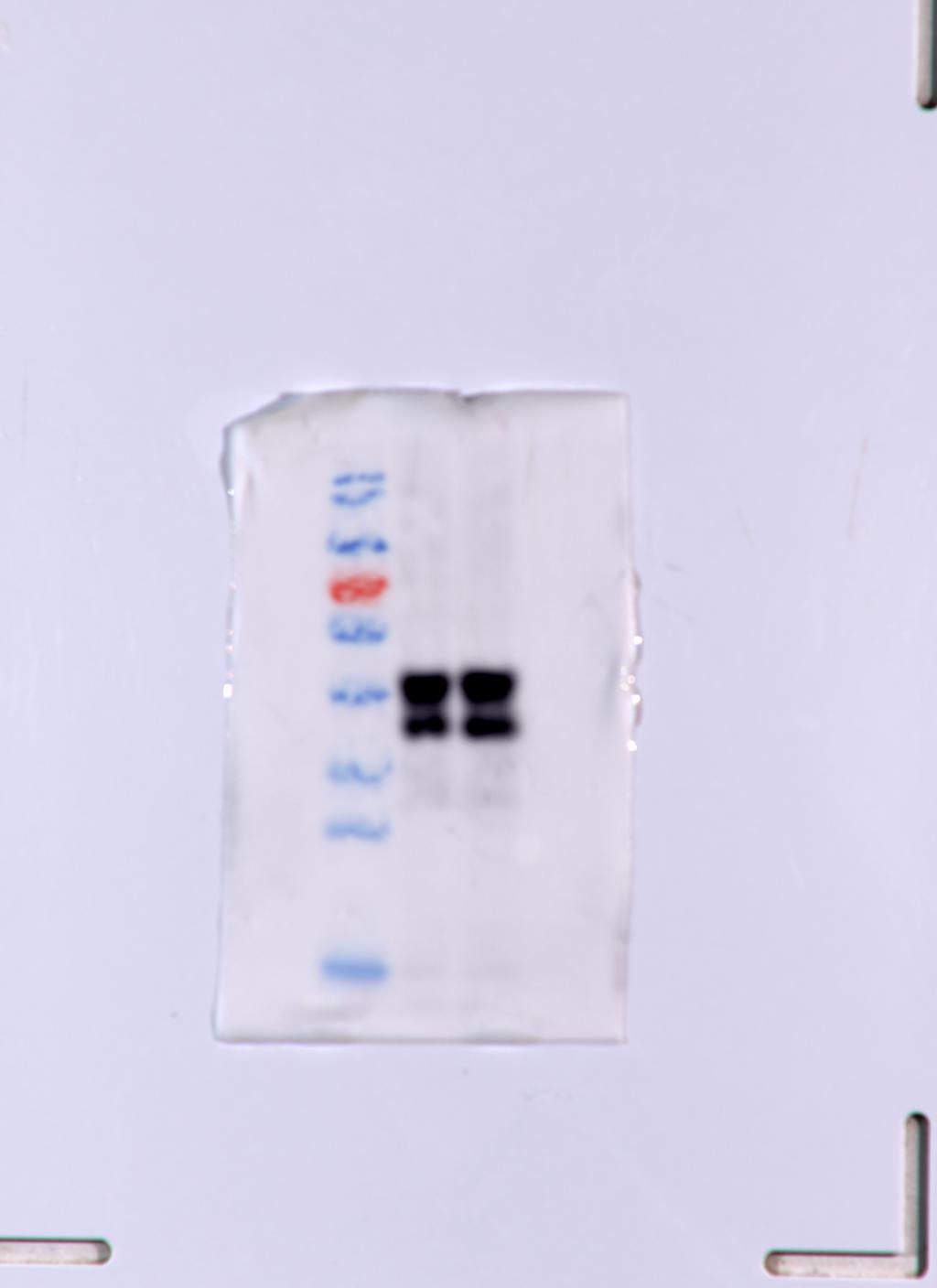

Supplement: Supplementary file 2 [file Data_Sheet_2.zip › Figure 5/MHC-I GAPDH.jpg]

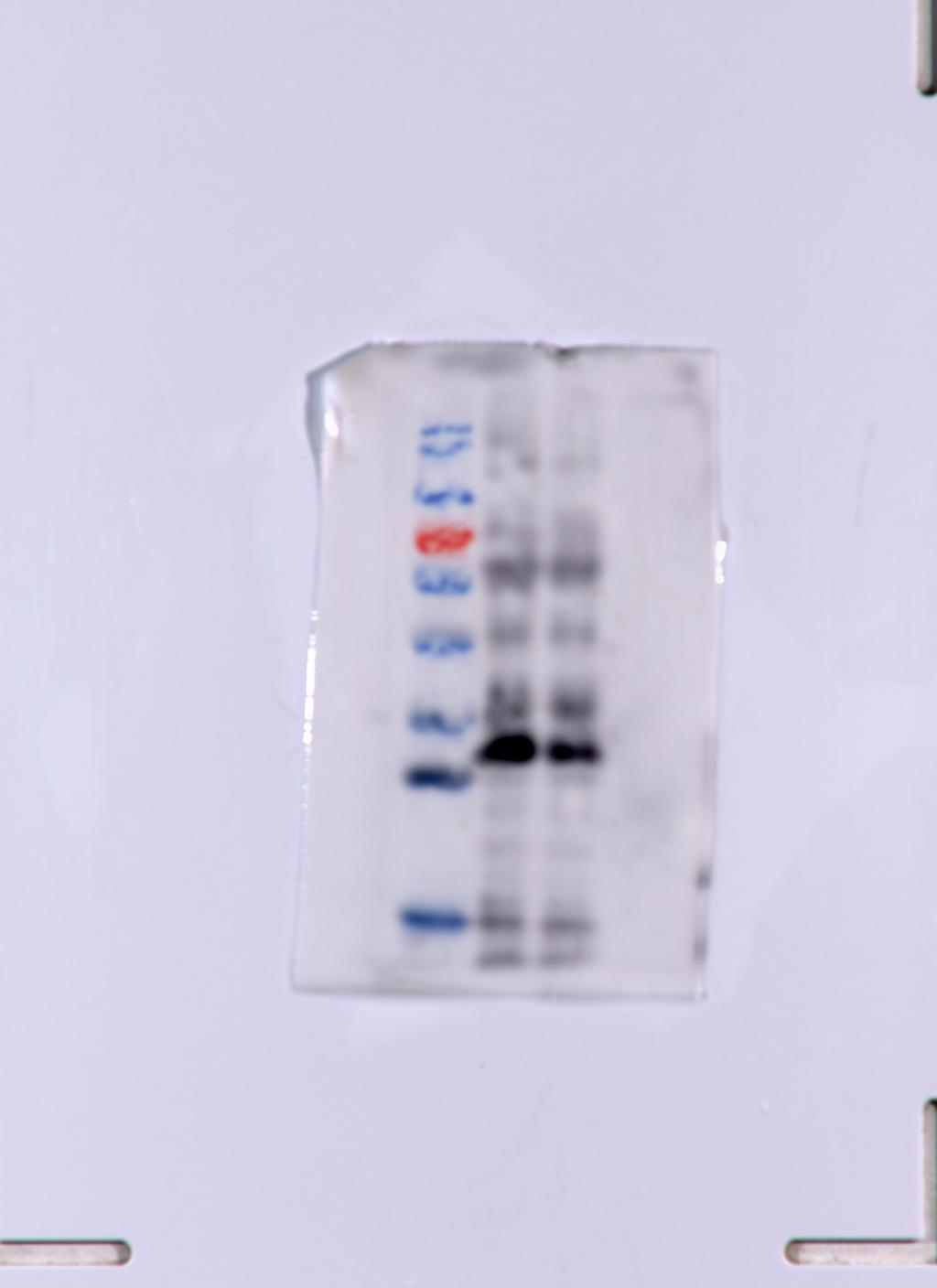

Supplement: Supplementary file 2 [file Data_Sheet_2.zip › Figure 5/MHC-I.jpg]
